# Enzymatic bypass and the structural basis of miscoding opposite the DNA adduct 1,*N*^2^-ethenodeoxyguanosine by human DNA translesion polymerase η

**DOI:** 10.1016/j.jbc.2021.100642

**Published:** 2021-04-08

**Authors:** Pratibha P. Ghodke, Jyotirling R. Mali, Amritraj Patra, Carmelo J. Rizzo, F. Peter Guengerich, Martin Egli

**Affiliations:** 1 Department of Biochemistry, School of Medicine, Vanderbilt University, Nashville, Tennessee, USA; 2 Department of Chemistry, College of Arts and Science, Vanderbilt University, Nashville, Tennessee, USA; 3 Vanderbilt-Ingram Cancer Center, Nashville, Tennessee, USA

**Keywords:** DNA polymerase, X-ray crystallography, mass spectrometry, DNA damage, DNA replication, DNA–protein interaction, translesion synthesis, etheno DNA adducts, 1,*N*^2^-ε-G, 1,*N*^2^-ethenodeoxyguanosine or 1,*N*^2^-ethenoguanine, CID, collision-induced dissociation, dAMPnPP, 2′-deoxyadenosine-5′-[(α,β)-imido]triphosphate, dCMPnPP, 2′-deoxycytosine-5′-[(α,β)-imido]triphosphate, dNMPnPP, 2′-deoxynucleoside-5′-[(α,β)-imido]triphosphate, EIC, extracted ion chromatogram, ESI, electrospray ionization, FAM, 6-carboxyfluorescein, h, human, pol, DNA polymerase, UDG, uracil DNA glycosylase, UPLC, ultraperformance liquid chromatography

## Abstract

Etheno (ε)-adducts, *e.g.*, 1,*N*^2^-ε−guanine (1,*N*^2^-ε-G) and 1,*N*^6^-ε−adenine (1,*N*^6^-ε-A), are formed through the reaction of DNA with metabolites of vinyl compounds or with lipid peroxidation products. These lesions are known to be mutagenic, but it is unknown how they lead to errors in DNA replication that are bypassed by DNA polymerases. Here we report the structural basis of misincorporation frequencies across from 1,*N*^2^-ε-G by human DNA polymerase (hpol) η. In single-nucleotide insertions opposite the adduct 1,*N*^2^-ε-G, hpol η preferentially inserted dGTP, followed by dATP, dTTP, and dCTP. This preference for purines was also seen in the first extension step. Analysis of full-length extension products by LC-MS/MS revealed that G accounted for 85% of nucleotides inserted opposite 1,*N*^2^-ε-G in single base insertion, and 63% of bases inserted in the first extension step. Extension from the correct nucleotide pair (C) was not observed, but the primer with A paired opposite 1,*N*^2^-ε-G was readily extended. Crystal structures of ternary hpol η insertion-stage complexes with nonhydrolyzable nucleotides dAMPnPP or dCMPnPP showed a *syn* orientation of the adduct, with the incoming A staggered between adducted base and the 5’-adjacent T, while the incoming C and adducted base were roughly coplanar. The formation of a bifurcated H-bond between incoming dAMPnPP and 1,*N*^2^-ε-G and T, compared with the single H-bond formed between incoming dCMPnPP and 1,*N*^2^-ε-G, may account for the observed facilitated insertion of dGTP and dATP. Thus, preferential insertion of purines by hpol η across from etheno adducts contributes to distinct outcomes in error-prone DNA replication.

Exocyclic etheno (ε) nucleobase adducts, *e.g.*, 1,*N*^6^-ε-A, 3,*N*^4^-ε-C, *N*^2^,3-ε-G, and 1,*N*^2^-ε-G (1, 2), arise from the action of various *bis*-electrophiles with cellular DNA (3) (Fig. 1). Reactive species that generate such lesions and originate from exogenous sources include epoxides derived from the oxidation of vinyl chloride and other vinyl monomers (4, 5) (Fig. 2*A*), urethane, certain nitrosamines (6), and mucochloric acid (7). In addition, etheno lesions are formed endogenously through interaction of nucleobases with lipid peroxidation-derived aldehydes and hydroxyalkenals (8, 9, 10) (Fig. 2*B*). Thus, etheno-DNA lesions have been detected in the human livers with nonalcoholic fatty liver disease, a cause of end-stage liver disease and hepatocellular carcinoma (11). Reactive oxygen species constitute an important factor in hepatocellular carcinoma pathogenesis and can react with polyunsaturated fatty acids that are derived from membrane phospholipids, *e.g.*, aldehydes such as 2,3-epoxy-4-hydroxynoneal and 4-hydroperoxynonenal react with DNA to form exocyclic etheno adducts (8, 10, 11). Other potential sources of ROS are inflammatory processes and cytochrome P450 (P450) 2E1. However, it has been established that hepatic etheno DNA adducts correlated significantly with 4-hydroxynonenal but not with levels of P450 2E1 (12).

**Figure 1:**
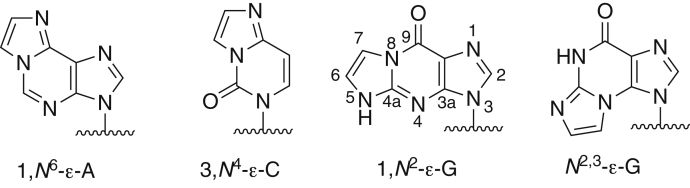
**Major etheno (ε) adducts.** The numbering pattern is shown for 1,*N*^2^-ε-G.

**Figure 2:**
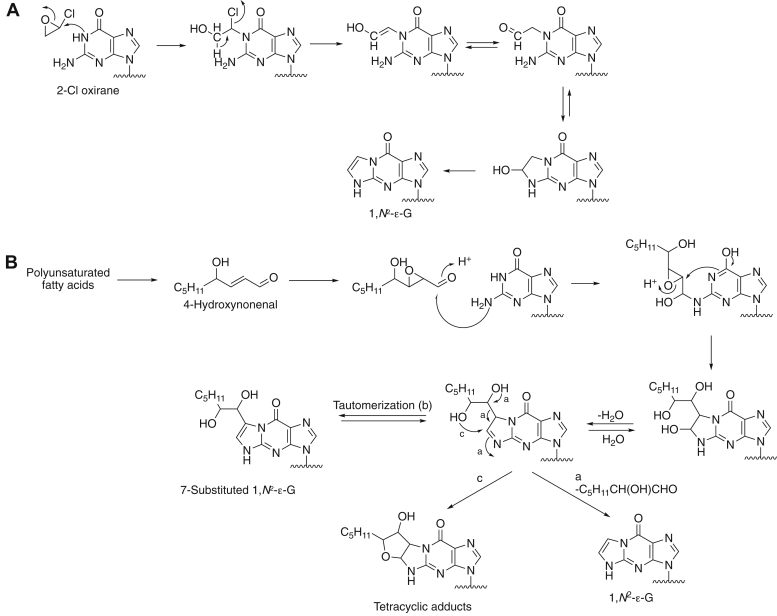
**Mechanisms of formation of 1,*N***^**2**^**-ε-G in DNA and RNA.***A*, reaction with 2-chlorooxirane (vinyl chloride epoxide), based on ^13^C labeling (5); *B*, reaction of the lipid peroxidation product 4-hydroxynonenal (11).

1,*N*^2^-ε-G was first synthesized in 1977 (13), and the 1,*N*^2^-ε-G base was later found to be excreted in human urine, indicating its presence in cellular DNA and removal by glycosylases (14). 1,*N*^2^-ε-G caused misincorporation and mutations with model DNA polymerases (pols) *in vitro* (*e.g.*, G→T and G→C base changes) and in bacterial (G→T, G→C and G→A base changes in *Escherichia coli*) and mammalian cells (G→A and G→T base changes) (15, 16, 17). However, the mutation rates following introduction of the 1,*N*^2^-ε-G lesion into bacterial or mammalian cell lines were relatively low (16, 17), a finding that was subsequently attributed to some degree to excision of the lesioned base by glycosylases such as *E. coli* uracil DNA glycosylase (UDG) and human alkyl-*N*-purine DNA glycosylase (18). These results leave open the question of the mechanism of miscoding and the potential involvement of a wider spectrum of pols beyond the replicative enzymes initially tested. The relevance of the etheno adducts in mutagenesis and carcinogenesis (1, 19, 20, 21) provided a motivation to more closely examine the consequences of these lesions for DNA damage-inducible pols. The 1,*N*^2^-ε-G adduct in which the two exocyclic carbon atoms prevent the formation of normal Watson–Crick H-bonds served as a useful model system for other exocyclic G lesions in such pol structure/function investigations (3, 22, 23).

In addition to the high-fidelity replicative pols, eubacteria, eukaryotes, and archaebacteria all have at their disposal the so-called translesion synthesis (TLS) or bypass polymerases (pols) (24, 25, 26, 27, 28, 29). These Y-Family pols play major roles in replication past DNA damage, whereby TLS can proceed in both an error-prone and an error-free fashion. Y-family pols share the right-handed palm, finger, and thumb domains with replicative ones but feature an additional little finger domain and unique makeups of their active sites. *E. coli* possesses five pols, with pols IV and V being members of the Y-Family (30). Humans have at least 19 pols, of which pols η, κ, and ι and Rev1 belong to the Y-Family. In the model archaeal organism *Sulfolobus solfataricus*, DNA pol IV (Dpo4) exhibits bypass behavior that is in some cases similar to that of human pol η (29). In initial work directed at the activity of a bypass pol past the 1,*N*^2^-ε-G adduct, we investigated the steady-state kinetics of Dpo4-catalyzed insertion and extension reactions (31). Experiments with individual dNTPs showed that A was the base preferentially inserted opposite 1,*N*^2^-ε-G. The products from full-length extension reactions were identified by an LC-MS/MS approach that revealed Dpo4 used multiple bypass mechanisms. Dpo4 extended primers containing 1,*N*^2^-ε-G to full length such that frameshifts predominated over A incorporation opposite the lesion. Thus, for a 3′-(1,*N*^2^-ε-G)CACT-5′ template, 5′-GTGA-3′ (frameshift, no insertion opposite 1,*N*^2^-ε-G) was the major extension product and 5′-AGTGA-3′ was a minor product. Generation of the former entails a frameshift such that primer G base pairs with template C. Crystal structures of a binary Dpo4-DNA and three ternary Dpo4-DNA-ddNTP complexes revealed an *anti* conformation of 1,*N*^2^-ε-G that was stacked between adjacent base pairs but unopposed by a residue from the primer strand (31). In these so-called Type 2 structures, Dpo4 is able to accommodate two template bases in the active site, thereby trapping a frameshift intermediate and shedding light on the mechanism of incorporation into the growing primer of either A or G opposite T or C, respectively, located on the 5′-side of the lesion.

A subsequent study investigated the abilities of four human pols to synthesize past the 1,*N*^2^-ε-G adduct and their tendencies for error-prone or error-free bypass (32). One of them, the replicative human pol δ was completely blocked by the etheno lesion. The human Y-family pols κ and ι exhibited similar rates of either dTTP or dCTP incorporation. In comparison, hpol η was more efficient at bypassing the lesion than hpols κ and ι, but it also showed the highest error frequency, incorporating dGTP > dATP > dCTP. This order was maintained irrespective of whether the 5′-adjacent residue to the lesion was C or T. Therefore, Dpo4 and hpol η use different mechanisms of bypassing the 1,*N*^2^-ε-G lesion, whereby the latter pol appears to consistently insert a base opposite 1,*N*^2^-ε-G and then extend the primer in an error-free fashion.

In the present work, we conducted further insertion and extension experiments with the most active of the human bypass pols, pol η, opposite the 1,*N*^2^-ε-G lesion. The bypass behavior was assessed both with the 18mer 5′-d(CAT [1,*N*^2^-ε-G]AT GAC GCT TCC CCC)-3′ (T(εG)A, **1**) and 5′-d(TCA T[1,*N*^2^-ε-G]G AAT CCT TCC CCC)-3′ (T(εG)G, **2**) template strands, positioning the lesion between A and T and G and T, respectively. hpol η favored insertion of dGTP opposite the adduct in both sequence contexts, followed by dATP ≅ dTTP > dCTP for the 5′-T(εG)A-3′ (**1**) template and dATP > dTTP > dCTP for the 5′-T(εG)G-3′ (**2**) template. The ability of hpol η to extend from the lesion was tested after the correct (1,*N*^2^-ε-G:C) base pair and a (1,*N*^2^-ε-G:A) mispair. Interestingly, the polymerase was unable to extend from the former but capable of carrying out postlesion extension from the latter.

We determined crystal structures of two insertion-stage hpol η complexes with the template/primer duplex 5′-d(CAT [1,*N*^2^-ε-G]AT GAC GCT)-3′/3′-d(TA CTG CGA)-5′ and either incoming dAMPnPP or dCMPnPP (nonhydrolyzable analogs of dATP and dCTP, respectively) in the presence of Mg^2+^. The incoming dAMPnPP adopts a staggered orientation opposite 1,*N*^2^-ε-G in the *syn* orientation, with H-bond formation between the N6 atom of A and both the O6 atom of 1,*N*^2^-ε-G and the O4 atom of the 5'-T. In the complex with incoming dCMPnPP, the lesion also assumes the *syn* orientation but pairs opposite C with formation of an H-bond between the N4 atom of C and the O6 atom of 1,*N*^2^-ε-G.

The combined hpol η bypass and structural data with DNA template–primer duplexes containing the 1,*N*^2^-ε-G adduct contribute to a better understanding of the diverse mechanisms underlying the preferred insertion of dATP and dGTP by this pol opposite distinct lesions. In previous work directed at hpol η bypass of abasic sites and the 1,*N*^6^-ε-A adduct, we established the preferential insertion of dATP and dGTP opposite these lesions (33, 34), as seen here with the bypass of 1,*N*^2^-ε-G. However, structures of insertion stage complexes reveal that the “purine rule” with an abasic site involves interactions between the incoming nucleotide base and the template strand backbone that can also result in frameshifts (33). Both incoming dGTP and dATP stack onto the 1,*N*^6^-ε-A adduct, which adopts the *anti* conformation at the hpol η active site, whereas incoming dTTP is coplanar with the lesion in the *syn* conformation. Conversely, the preferred insertion of dGTP and dATP by hpol η occurs with the 1,*N*^2^-ε-G lesion in the *syn* conformation without the involvement of stacking between the lesioned and incoming nucleotide base moieties. 1,*N*^2^-ε-G maintains the *syn* orientation even opposite incoming dCTP, and unlike the case of bypass of abasic sites, the preferred insertion of purine nucleotides opposite 1,*N*^2^-ε-G is devoid of frameshifts.

## Results

### Translesion synthesis across from the 1,*N*^2^-ε-G adduct by hpol η

**Figure 3:**
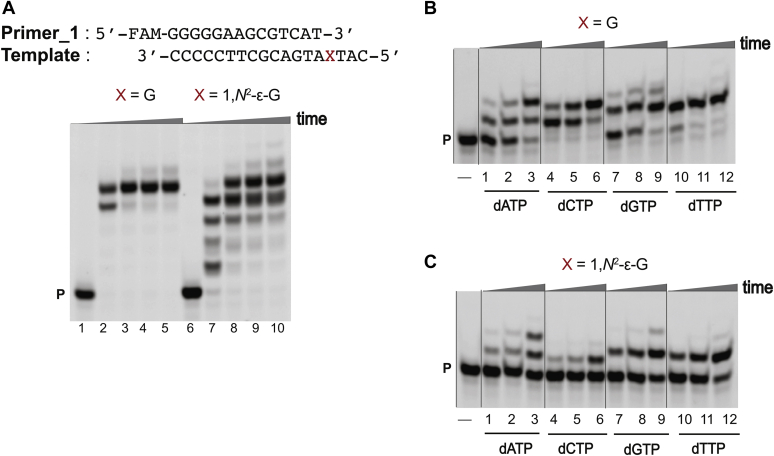
**hpol η-mediated bypass across from 1,*N***^**2**^**-ε-G in template 5′-T(εG)A-3′ (1).** PAGE: 20%, 7 M urea. *A*, full-length extension assay: hpol η (120 nM) elongated Primer_1 opposite G, and 1,*N*^2^-ε-G-containing DNA templates in the presence of a mixture of dNTPs (500 μM). All reactions were done at 37 °C for 5-, 30-, 60-, and 120-min (time gradients indicated with wedges). Lanes: 1 to 5 for unmodified template; 6 to 10 for 1,*N*^2^-ε-G-modified template. Single-nucleotide insertion assays: hpol η (10 nM) was incubated with *B*, Primer_2/control template 1, and *C*, Primer_2/1,*N*^2^-ε-G modified template 1 (5′-T(εG)A-3′) as well as individual dNTPs (100 μM). Lanes: 1 to 3 for dATP, 4 to 6 for dCTP, 7 to 9 for dGTP, 10 to 12 for dTTP. All reactions were done at 37 °C for 5-, 10-, and 30-min. P indicates the FAM-labeled Primer_1. See Experimental procedures and Table S1 for the oligonucleotide sequences used.

hpol η-mediated bypass of 1,*N*^2^-ε-G was performed using two different sequence contexts, 5′-T(εG)A-3′ (**1**) and 5′-T(εG)G-3′ (**2**) (see Table S1 for oligonucleotide sequences), employing full-length extension and single-nucleotide insertion assays. The oligonucleotide sequence context 5′-T(εG)A-3′ (**1**) was designed on the basis of the sequence used for crystallography. The 5′-T(εG)G-3′ (**2**) oligonucleotide was studied previously (32). Full-length primer extension reactions were performed in the presence of all four dNTPs using the respective primers (see Table S1 for oligonucleotide sequences). Under our experimental conditions, the bypass of 1,*N*^2^-ε-G was slower compared with unmodified templates (Fig. 3*A* and Fig. S4*A*) for both sequence contexts.

Single-nucleotide insertion assays were done utilizing individual dNTPs to determine the insertions across the adduct. The incorporation preference for the control templates was dCTP > dTTP > dGTP > dATP (Fig. 3*B*, Fig. S4*B* lanes 1–12). hpol η preferentially added dGTP across from 1,*N*^2^-ε-G, at almost similar rates for both sequence contexts. The incorporation preference for the 5′-T(εG)A-3′ (**1**) template was dGTP > dATP ≅ dTTP > dCTP (Fig. 3*C*, lanes 1–12) while for the 5′-T(εG)G-3′ (**2**) template, the preference was dGTP > dATP > dTTP > dCTP (Fig. S4*C*, lanes 1–12). These results show that hpol η tolerates 1,*N*^2^-ε-G in both sequence contexts.

### Postlesion extension past the 1,*N*^2^-ε-G adduct by hpol η

hpol η-mediated postlesion extension for 1,*N*^2^-ε-G was performed for both the sequence contexts 5′-T(εG)A-3′ (**1**) and 5′-T(εG)G-3′ (**2**) (see Table S1 for oligonucleotide sequences) employing full-length extension and single-nucleotide insertion assays. We utilized two different primers for postlesion extension after the correct base pair (1,*N*^2^-ε-G:C) and a mispair (1,*N*^2^-ε-G:A). For a misincorporated base, A was used opposite the lesion because it showed the greatest misincorporation frequency, following that for dGTP.

**Figure 4:**
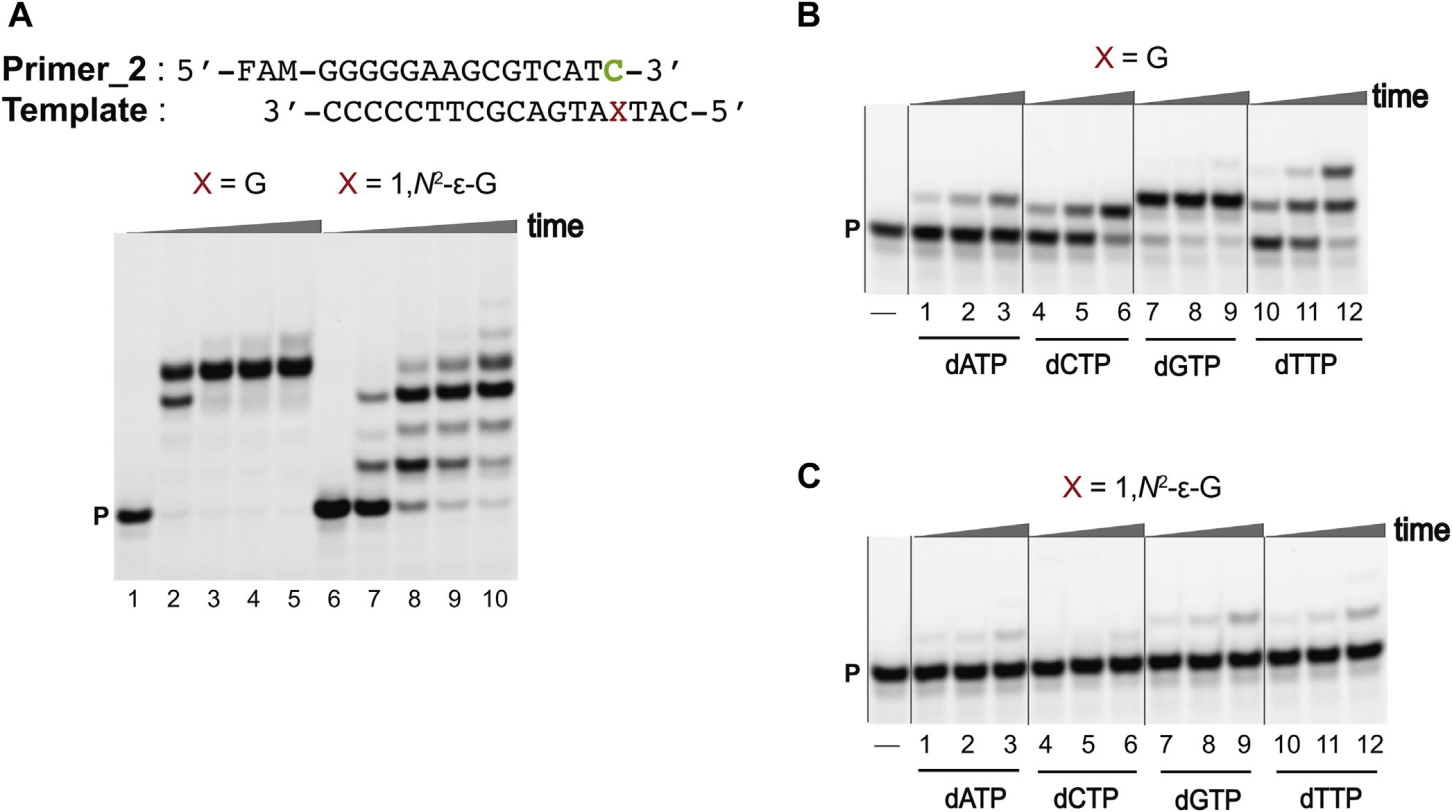
**hpol η-mediated postlesion full-length and single-nucleotide insertion assays using 1,*N***^**2**^**-ε-G in template 5′-T(εG)A-3′ (1) and Primer_2.** PAGE (20%, 7 M urea): *A*, full-length extension assay: hpol η (120 nM) elongated Primer_2 opposite G, and 1,*N*^2^-ε-G-containing DNA templates in the presence of a mixture of dNTPs (500 μM). All reactions were done at 37 °C for 5-, 30-, 60-, and 120-min (time gradients indicated with wedges). Lanes: 1 to 5 for unmodified template; 6 to 10 for 1,*N*^2^-ε-G-modified template. Single-nucleotide insertion assays: hpol η (10 nM) was incubated with *B*, Primer_2/control template 1, and *C*, Primer_2/1,*N*^2^-ε-G modified template 1 (5′-T(εG)A-3′), as well as individual dNTPs (100 μM). Lanes: 1 to 3 for dATP, 4 to 6 for dCTP, 7 to 9 for dGTP, 10 to 12 for dTTP. All reactions were done at 37 °C for 5-, 10-, and 30-min. P indicates the FAM-labeled Primer_2. See Experimental procedures and Table S1 for the oligonucleotide sequences used.

**Figure 5:**
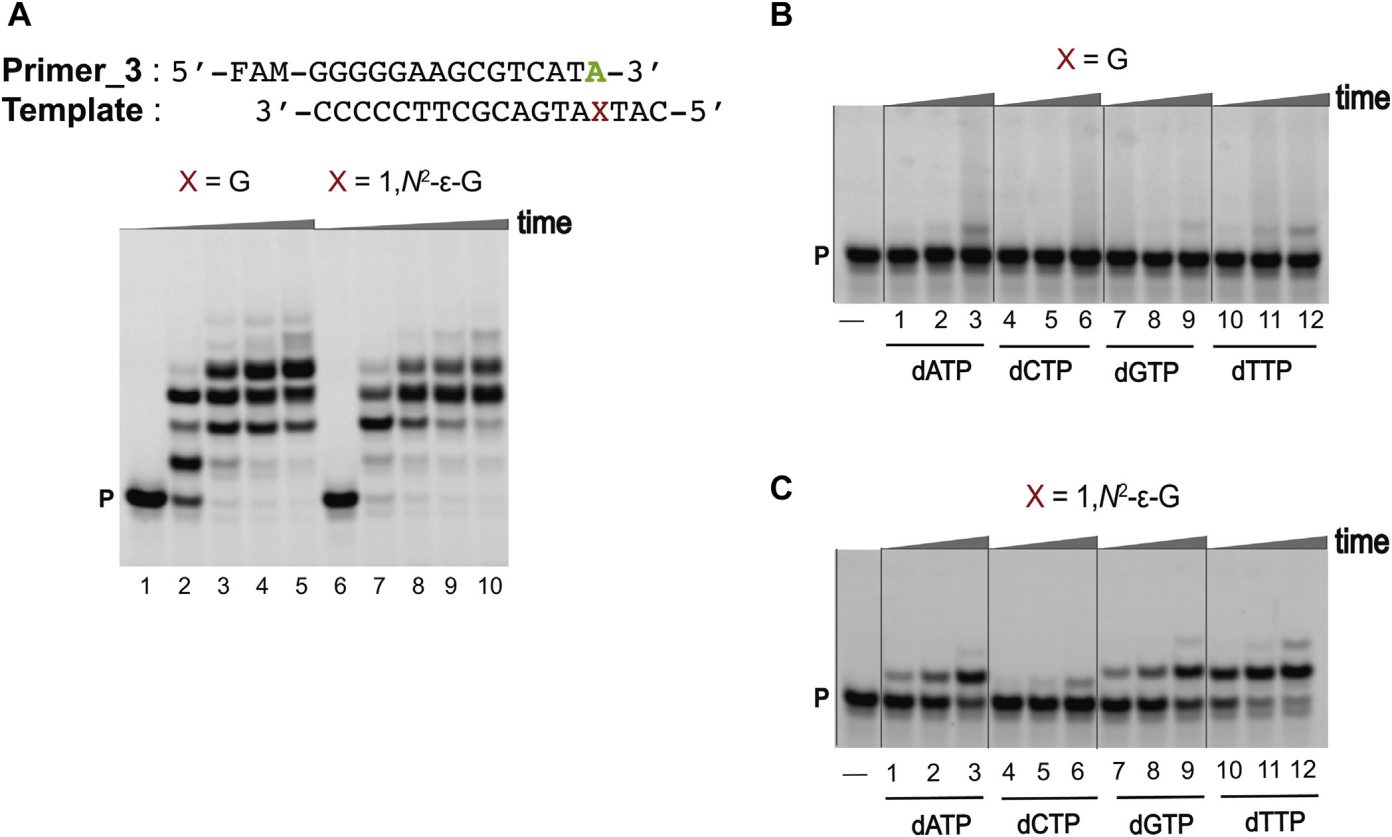
**hpol η-mediated postlesion full-length and single-nucleotide insertion assays using 1,*N***^**2**^**-ε-G in template 5′-T(εG)A-3′ (1) and Primer_3.** PAGE (20%, 7 M urea). *A*, full-length extension assay: hpol η (120 nM) elongated Primer_3 opposite G, and 1,*N*^2^-ε-G-containing DNA templates in the presence of a mixture of dNTPs (500 μM). All reactions were done at 37 °C for 5-, 30-, 60-, and 120-min (time gradients indicated with wedges). Lanes: 1 to 5, unmodified template; 6 to 10, 1,*N*^2^-ε-G-modified template. Single-nucleotide insertion assays: hpol η (10 nM) was incubated with *B*, Primer_2/control template 1, and *C*, Primer_3/1,*N*^2^-ε-G modified template 1 (5′-T(εG)A-3′), as well as individual dNTPs (100 μM). Lanes: 1 to 3 for dATP, 4 to 6 for dCTP, 7 to 9 for dGTP, 10 to 12 for dTTP. All reactions were done at 37 °C for 5-, 10-, and 30-min. P indicates the FAM-labeled Primer_3. See Experimental procedures and Table S1 for the oligonucleotide sequences used.

Full-length primer extension reactions were performed in the presence of all four dNTPs using the respective primers (see Table S1 for oligonucleotide sequences). As a result, for both the sequence contexts, under our experimental conditions, extension from the 1,*N*^2^-ε-G:C pair was slower (Fig. 4*A* and Fig. S5*A*, lane 7) compared with the unmodified templates (Fig. 4*A* and Fig. S5*A*, lane 2). For the 1,*N*^2^-ε-G:A pair, extension was faster (Fig. 5*A* and Fig. S6*A*, lane 7) compared with unmodified templates (Fig. 5*A* and Fig. S6*A*, lane 2).

Single-nucleotide insertion assays were done utilizing individual dNTPs to analyze postlesion insertions after the correct base pair (1,*N*^2^-ε-G:C) as well as mispairing (1,*N*^2^-ε-G:A).

For correct base pairing, the incorporation preference for control template 1 was dTTP > dGTP > dCTP > dATP (Fig. 4*B*, lanes 1–12), and the incorporation preference for the control template 2 was dATP > dTTP >dGTP > dCTP (Fig. S5*B* lanes 1–12). In the case of the 1,*N*^2^-ε-G:C template–primer pairs, postlesion insertion was drastically retarded, indicating resistance to extension past the correct base pair C. Overall, hpol η-mediated formation of very small amounts of inserted products for both sequence contexts was observed, at similar rates. The postlesion insertion preference for 5′-T(εG)A-3′ (**1**) template was dTTP > dGTP > dATP > dCTP (Fig. 4*C*, lanes 1–12) while for the 5′-T(εG)G-3′ (**2**) template, the preference was dGTP > dATP ≅ dTTP ≅ dCTP (Fig. S5*C*, lanes 1–12).

For a mispair (G:A), postlesion insertion was drastically affected for the control template 1 (Fig. 5*B*, lanes 1–12), while the incorporation preference for the control template 2 was dTTP > dATP ≅ dGTP > dCTP (Fig. S6*B*, lanes 1–12). In the case of the 1,*N*^2^-ε-G:A template–primer pairs, the postlesion insertions were unaffected (Fig. 5*C*, Fig. S6*C*). The order of postlesion insertion for both the sequence contexts was dTTP > dGTP > dATP > dCTP (Fig. 5*C*, Fig. S6*C*, lanes 1–12). The preference for T insertion during hpol η-mediated postlesion bypass was also observed previously for unmodified (2′-F-G:C pair) as well as modified (*N*^7^-CH_3_ 2′-F-G:C pair) templates (35).

Overall, hpol η was unable to perform postlesion extension for the correct pair (1,*N*^2^-ε-G:C). hpol η was able to perform postlesion extension for a mispair (1,*N*^2^-ε-G:A), indicating that the mispair was preferentially extended past the lesion.

### LC-ESI-MS/MS analysis for hpol η-mediated primer extension across from 1,*N*^2^-ε-G

To elucidate details about other possible miscoding events, hpol η-mediated full-length extension reactions were performed for both the sequence contexts, 5′-T(εG)A-3′ (**1**) and 5′- T(εG)G-3′ (**2**) for LC-MS/MS analysis (see Table S1 for oligonucleotide sequences). The 2′-deoxyuridine (dU)-containing primers (see Table S1 for oligonucleotide sequences) were used for the extension reaction. Next, the fully extended reaction mixtures were treated with UDG and piperidine as described earlier (31, 36). Reaction mixtures were also analyzed by gel electrophoresis to confirm the elongations (Fig. S7). The relative yields of fully extended products were calculated by LC-MS/MS analysis and are summarized in Table 1 and Table S6. CID spectra of the products are shown in Figs. S8–S19 and Tables S3–S10.

**Table 1:** Summary of products of extension of 1,*N*^2^-ε-G-modified template 5′-T(εG)A-3′ (1) and Primer_4 by hpol η analyzed by LC-ESI-MS/MS

Primer_4: 5’-FAM-GGGGGAAGCG**U**CAT-3’ Template: 3’-CCCCCTTCGCAGTA**X**TAC-5’				
X	Sequence	Yield	Observed *m/z* (charge)	Base added
G	5’-pCAT**C**ATGA-3’	70%	823.64 (−3)	C, plus, blunt end addition of A, and G
	5’-pCAT**C**ATGG-3’	30%	828.64 (−3)	
1,*N*^2^-ε-G	5′-pCAT**AG**TGA-3′	15%	836.64 (−3)	A, followed by misinsertion of G, plus, blunt end addition of A
	5′-pCAT**G**ATGA-3′	38%	836.64 (−3)	G, plus, blunt end addition of A
	5′-pCAT**GG**TGA-3′	47%	842.00 (−3)	G, followed by misinsertion of G plus, blunt end addition of A

Replication of the control templates gave only error-free products, with blunt end addition of A and G (Table 1 and Table S6).

**Figure 6:**
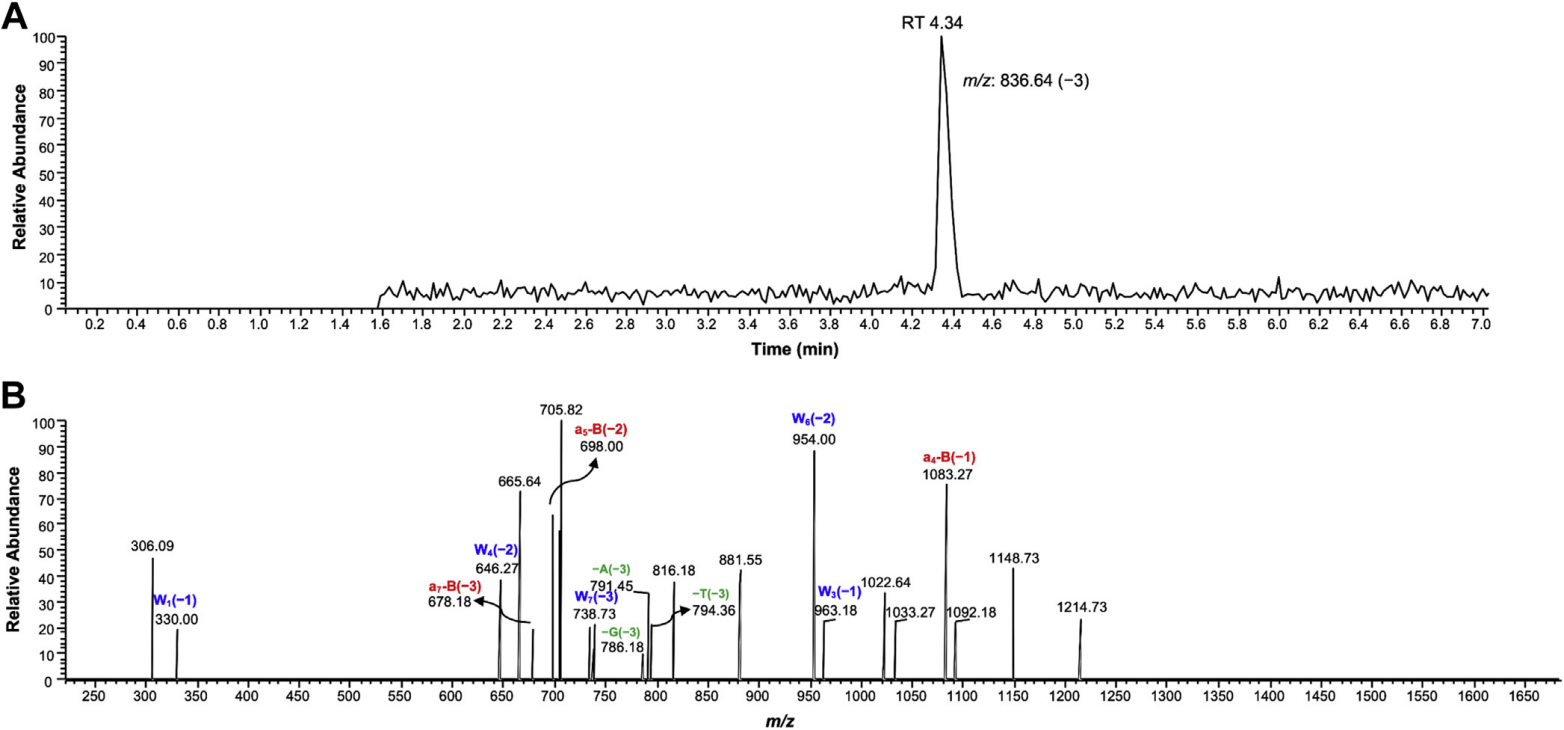
**Extracted ion chromatogram and CID spectrum of *m/z* 836.64 ion.***A*, chromatogram; *B*, mass spectrum. The *m/z* 836.64 ion (−3, *t*_R_ 4.34) is associated with the extended product sequence 5′-pCAT**AG**TGA-3′ for 1,*N*^2^-ε-G template 5′-T(εG)A-3′ (**1**)–Primer_4 complex. a-B fragments represented in *red*, W fragments in *blue*, and base losses in *green*.

In case of the 5′-T(εG)A-3′ (**1**) template, hpol η replicated past the adduct in an error-prone manner only, resulting in three main products (Table 1). The first two products showed the same *m/z* (*i.e.*, 836.64, −3 ion) and coeluted together at same retention time (*t*_R_ 4.34 min, Figs. S8 and S10). In these two products, the first product corresponded to misincorporation of A opposite the adduct, followed by misinsertion of G with blunt end addition of A (m/z 836.64, −3 ion: 5′-pCAT**AG**TGA-3′; 15%; Fig. 6 and Table S3), and the second product corresponded to misincorporation of G with blunt end addition of A (m/z 836.64, −3 ion: 5′-pCAT**G**ATGA-3′; 38%; Fig. S9 and Table S4). The last product corresponded to misincorporation of G opposite the adduct, followed by misinsertion of G and with blunt end addition of A (m/z 842.00, −3 ion (*t*_R_ 4.34 min): 5′-pCAT**GG**TGA-3′; 47%; Fig. S11 and Table S5). The insertion of G (opposite 1,*N*^6^-ε-A) followed by misinsertion of another G has been observed in previous studies (37).

For the 5′-T(εG)G-3′ (**2**) template, hpol η-mediated error-prone bypass resulted in four main products (Table S6). The first two products showed the same *m/z* (836.64, −3 ion) and coeluted at the same retention time (*t*_R_ 4.33 min, Figs. S12 and S15). Interestingly, the next two products also coeluted at same *t*_R_ and *m/z* (842.00, −3 ion, *t*_R_ 4.31 min, Figs. S16 and S19). Among all the products, the first corresponded to misincorporation of A opposite to the adduct with blunt end addition of G (m/z 836.64, −3 ion: 5′-pTC**A**ATGAG-3′; 20%; Fig. S13 and Table S7). The second product corresponded to misincorporation of G with blunt end addition of A (m/z 836.64, −3 ion: 5′-pTC**G**ATGAA-3′; 25%; Fig. S14 and Table S8). The third product corresponded to misincorporation of A followed by misinsertion of G, with misinsertion of G opposite the 5′ T and blunt end addition of A (m/z 842.00, −3 ion: 5′-pTC**AG**TG**G**A-3′; 26%; Fig. S17 and Table S9). The last product corresponded to misincorporation of G opposite the adduct, misinsertion of G opposite the 5′ T, with blunt end addition of A (m/z 842.00, −3 ion: 5′-pTC**G**ATG**G**A-3′; 29%; Fig. S18 and Table S10).

No products were observed containing the incorporation of correct base C (or its stalled products), indicating that correct base C can be inserted opposite but that hpol η is not able to extend the primer to obtain full-length products, as reflected in postlesion extension assays.

### Crystal structures of ternary hpol η insertion stage complexes with 1,*N*^2^-ε-G opposite dAMPnPP and dCMPnPP

We determined two crystal structures of hpol η⋅DNA (oligonucleotide)⋅dNTP ternary complexes trapped in the insertion state. Both complexes feature the same 8mer oligonucleotide primer and a 12mer oligonucleotide template with the 1,*N*^2^-ε-G adduct flanked by 5′-T and 3′-A (Table S2). The structure of the complex with incoming dAMPnPP was refined to 2.59 Å resolution, and the complex with incoming dCMPnPP was refined to 2.23 Å resolution. Selected crystal data, data collection, and refinement parameters are listed in Table 2. Examples of the quality of the electron density in the final models are depicted in Figs. S20 and S21. Along with the polymerase, oligonucleotide duplex, and nucleotide triphosphates, the dAMPnPP and dCMPnPP structures revealed 163 and 273 water molecules, respectively, and two active site Mg^2+^ ions. dAMPnPP and dCMPnPP are nonhydrolyzable nucleotide triphosphates that allow cocrystallization of pols with oligonucleotide template–primer constructs in the presence of Mg^2+^ without the use of a primer with a 3′-terminal 2′,3′-dideoxynucleoside and/or divalent metal ions that are not catalytically competent.

**Table 2:** Crystal data, data collection parameters, and structure refinement statistics^a^

Complex	1,*N*^2^-ε-G:dAMPnPP	1,*N*^2^-ε-G:dCMPnPP
Data Collection		
Wavelength [Å]	0.97856	0.97856
Space group	*P*6_1_	*P*6_1_
Resolution [Å]	50.0–2.59 (2.63–2.59)^a^	42.9–2.23 (2.29–2.23)^a^
Unit cell *a*, *b*, *c* [Å]	99.11, 99.11, 81.86	99.08, 99.08, 81.89
Unique reflections	14,272 (713)	22,384 (1659)
Completeness [%]	99.7 (100)	100 (100)
I/σ(I)	12.0 (1.8)	16.6 (2.9)
Wilson B-factor [Å^2^]	22.1	26.9
R-merge	0.152 (0.910)	0.093 (0.625)
Redundancy	5.7 (5.7)	5.7 (5.7)
Refinement		
R-work	0.191 (0.235)	0.158 (0.193)
R-free	0.248 (0.301)	0.210 (0.280)
Number of atoms		
Protein/DNA	3426/391	3394/391
dNTP/Water/Mg^2+^	30/163/2	28/273/2
Protein residues	431	430
B-factor [Å^2^]		
Average	38.6	33.5
Protein/DNA	37.8/45.9	32.6/37.5
dNTP/M^2+^/Water	41.3/33.4/38.7	34.4/26.5/38.5
R.m.s. deviations		
Bonds [Å]	0.003	0.008
Angles [deg.]	0.6	1.0
Ramachandran		
Favored (%)	96.8	97.0
Allowed (%)	3.0	2.8
Outliers (%)	0.2	0.2
PDB ID Code	5F9I	5F9N

a Statistics for the highest-resolution shell are shown in parentheses.

**Figure 7:**
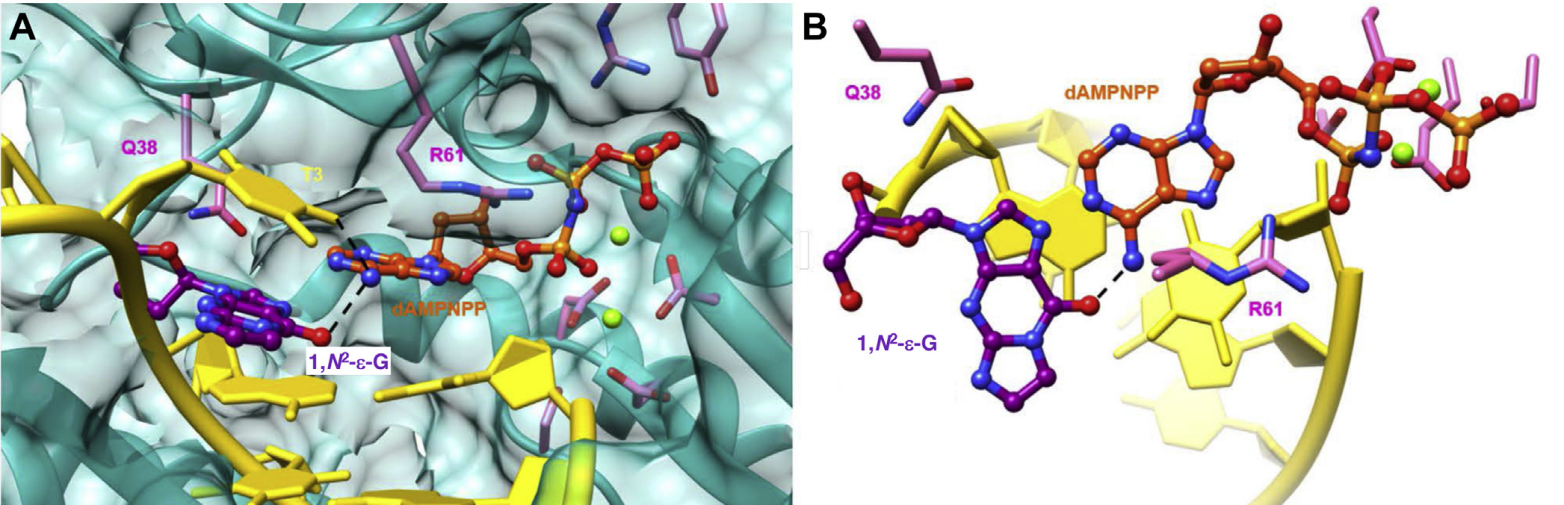
**Active site conformation in the ternary hpol η insertion step complex with dAMPnPP opposite 1,*N***^**2**^**-ε−G in the 5′-T(εG)A-3′ template sequence context (oligonucleotide 1).***A*, view into the DNA major groove. *B*, rotated by 90° and viewed perpendicular to the adenine and adduct base planes. Selected active site residues are colored by atom with carbon atoms shown in *purple* (1,*N*^2^-ε−G), *orange* (incoming dAMPnPP), or *pink* (Arg-61 and Gln-38 from the finger domain and Asp/Glu coordinating Mg^2+^ ions that are shown as *light green spheres*). The remaining template and primer residues are colored in *yellow* and H-bonds involving the incoming nucleotide are drawn with *dashed lines*.

**Figure 8:**
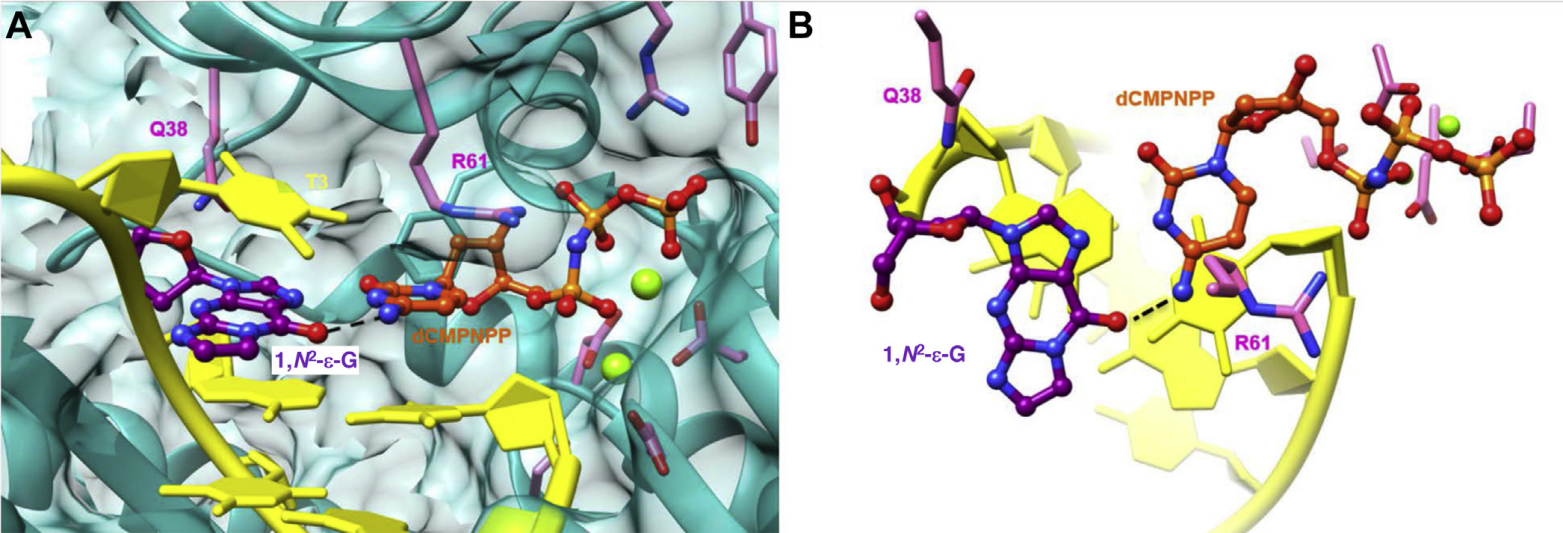
**Active site conformation in the ternary hpol η insertion step complex with dCMPnPP opposite 1,*N***^**2**^**-ε−G in the 5′-T(εG)A-3′ template sequence context (oligonucleotide 1).***A*, view into the DNA major groove. *B*, rotated by 90° and viewed perpendicular to the cytosine and adduct base planes. Selected active site residues are colored by atom with carbon atoms shown in *purple* (1,*N*^2^-ε−G), *orange* (incoming dCMPnPP), or *pink* (Arg-61 and Gln-38 from the finger domain and Asp/Glu coordinating Mg^2+^ ions that are shown as *light green spheres*). The remaining template and primer residues are colored in *yellow* and H-bonds involving the incoming nucleotide are drawn with *dashed lines*. It is unlikely that N1 of 1,*N*^2^-ε−G and N3 of dCMPnPP are H-bonded because the pH of the crystallization solution is too high for cytosine to be protonated at N3 and a tautomeric form of the adduct with the hydrogen on N1 cannot be invoked.

At the active sites of both complexes, 1,*N*^2^-ε-G adapts a *syn* orientation with the lesioned Watson–Crick edge of G jutting into the major groove (Figs. 7 and 8). The nucleobase of the incoming dAMPnPP is staggered relative to the 1,*N*^2^-ε-G base plane and sits halfway between the adduct and the 5′-adjacent thymine (Fig. 7*A*). This structure positions the N6 atom of adenine 2.9 Å from O6 of the adduct and 2.5 Å from O4 of thymine, consistent with the formation of two H-bonds. The projection, approximately perpendicular to the 1,*N*^2^-ε-G and adenine planes, shows that there is no stacking between the incoming nucleotide and the adduct (Fig. 7*B*). The 3′-hydroxyl group of the primer 3′-terminal dT is poised for attack at the α-phosphate of dAMPnPP with an O3’^…^Pα distance of 3.7 Å. Among hpol η side chains that project into the active site, Arg-61 plays a key role in that it is often engaged in multiple interactions with nucleobase atoms and/or phosphate groups of the incoming nucleotide (38, 39). In the case of the ternary complex with dAMPnPP, the guanidino moiety of arginine does not stack on adenine or engage in contacts with the Hoogsteen edge of the base, as Arg-61 is shifted slightly into the major groove vis-à-vis the incoming nucleotide (Fig. 7*B*). However, Arg-61 forms a somewhat long (3.4 Å) salt bridge with the α-phosphate and a water-mediated interaction with the γ-phosphate. Another amino acid side chain, Gln-38, that is positioned near the minor groove and occasionally engages in direct or water-mediated contacts with template base and/or incoming nucleotide (38, 39), is too far removed from both in the active site of this complex and does not form any with either.

Unlike dAMPnPP, the base moiety of the incoming dCMPnPP is coplanar with the base plane of 1,*N*^2^-ε-G (Fig. 8*A*). A single H-bond is formed between the N4 atom of cytosine and the O6 atom of the adduct. The O4 atom of the T 5′-adjacent to 1,*N*^2^-ε-G is too far removed from N4 of the incoming cytosine to engage in an H-bond interaction (3.5 Å). The 3′-hydroxyl group of the primer 3′-terminal T sits at a distance of 3.5 Å from the α-phosphate of dCMPNPP. As in the case of the complex with dAMPnPP, Arg-61 is shifted slightly into the major groove vis-à-vis the incoming cytosine (Fig. 8*B*), which precludes a stacking interaction. The guanidino moiety of Arg-61 is too far removed from the α-phosphate (4.1 Å) to engage in an effective interaction but forms a water-mediated contact with the γ-phosphate. In the minor groove, a water molecule bridges the Gln-38 side chain and O2 atom of the incoming dCMPnPP.

## Discussion

The Y-Family hpol η plays a key role in translesion synthesis in human cells and features an active site composition that can accommodate bulky adducts (24, 40). Among the best-known base lesions that hpol η can cope with are those that arise as a consequence of UV damage, *e.g.*, cyclic pyrimidine dimers, as well as cisplatin intrastrand cross-links (41). However, hpol η can also synthesize past a variety of other adducts with various proficiencies in a more or less error-prone fashion. Thus, hpol η can bypass the major oxidative lesion 8-oxoG efficiently and virtually error-free (42). Additional lesions that this polymerase can overcome include abasic sites (33), exocyclic etheno A and G adducts (32, 34), *O*^6^-methyl G (39), and *O*^4^-alkyl-T (43) as well as, in a remarkably efficient and mostly error-free mode, the methyl FAPY-G lesion (44), and a bulky aminobenzanthrone DNA adduct (45).

The insertion stage assays with both sequences, 5′-T(εG)A-3′ (**1**) and 5′-T(εG)G-3′ (**2**), demonstrate that hpol η preferentially inserts dGTP and dATP relative to the pyrimidine nucleoside triphosphates. In the case of sequence (**1**), dATP and dTTP were incorporated to a similar degree, but the correct dCTP nucleotide was the least preferred opposite 1,*N*^2^-ε-G in both sequence contexts (Fig. 3 and Fig. S4). The two crystal structures of hpol η ternary complexes reveal clear differences between the orientation of incoming dAMPnPP and dCMPnPP. The nucleobase of the former assumes a staggered orientation relative to the 1,*N*^2^-ε-G base plane and is thus positioned halfway between the adduct and template dT in the spacious active site of hpol η (Fig. 7). This allows formation of a bifurcated H-bond between incoming nucleotide (N6H_2_) and template dT (O4) and 1,*N*^2^-ε-G (O6). The overall preferred dGTP is likely to adopt a similar orientation and may use its N1H atom to establish a bifurcated H-bond to the O4 and O6 keto oxygens of dT and the adduct, respectively. Unlike an incoming dATP, dGTP features the exocyclic amino function in the minor groove that may also allow a direct or water-bridged H-bond interaction with the side chain of Gln-38. In the structure of the complex with incoming dCMPnPP, cytosine is virtually coplanar with the 1,*N*^2^-ε-G base plane, resulting in a single H-bond between N4H_2_ and the O6 keto oxygen of the adduct, but precluding an H-bond interaction with the 5′-adjacent template dT (Fig. 8). dTTP is preferentially inserted relative to dCTP in both sequence contexts and—assuming similar orientations of thymine and cytosine vis-à-vis 1,*N*^2^-ε-G—will likely establish an H-bond to the N7 atom of the adduct in the *syn* conformation *via* its N3 hydrogen donor function. As with an incoming dCMPnPP in the crystal structure, this pose would position the thymine O2 atom in the minor groove in the vicinity of the Gln-38 side chain so that the two could be linked by a bridging water molecule. Alternatively, it is possible that thymine shifts slightly toward the major groove, thus placing the N3 hydrogen donor roughly between the N7 and O6 acceptors of the adduct and potentially resulting in the formation of a bifurcated H-bond. The preferred insertion of dGTP and dATP by hpol η opposite 1,*N*^2^-ε-G is most likely a consequence of their ability to reach two template nucleotides, as stacking differences between incoming purine and pyrimidine nucleoside triphosphates do not appear to play a significant role in the observed discrimination. However, the staggered orientation of dAMPnPP (and presumably dGTP) does not result in frameshifting, and once inserted, the purine may settle opposite the adduct in a more or less coplanar fashion, thereby allowing hpol η to bypass 1,*N*^2^-ε-G without skipping a base.

Extension reactions were tested with primers that featured either A or C opposite the adduct. Not only was insertion of dCTP opposite the adduct disfavored relative to dGTP and dATP, but extension from the correct base opposite 1,*N*^2^-ε-G was severely hampered as well (Fig. 4*A* and Fig. S5*A*, lane 7). hpol η was able to extend from a G:A pair with one of the control templates and then inserted dTTP > dATP ≅ dGTP > dCTP, *i.e.*, the extension proceeded in an error-prone fashion. Conversely, extensions from 1,*N*^2^-ε-G:A proceeded more efficiently than the control reactions from G:A, and irrespective of the template sequence, hpol η inserted dTTP > dGTP > dATP > dCTP opposite T (Fig. 5 and Fig. S6). In a previous study from our laboratories (35), we observed that dTTP misincorporation was sixfold more efficient during hpol η-mediated postlesion bypass for the *N*^7^-CH_3_ 2′-F-G lesion compared with 2′-F-G unmodified template, when paired with the correct base C. In a similar way, hpol η catalyzed TLS past the 1,*N*^2^-ε-G adduct in an efficient but highly error-prone manner. The results of the full-length extension reactions analyzed by LC-MS/MS attest to the absence of frameshift products but are supportive of untemplated addition of A and G at the end of the primer. The polymerase preferentially incorporated dGTP opposite the adduct and in the first extension step (85% and 63%, respectively, Table 1).

In a previous study from our laboratories (32), we also reported that hpol η preferred to insert G and A opposite 1,*N*^2^-ε-G, with some C insertion. LC-MS extension analysis, with what is referred to here as oligonucleotide **2**, yielded only the product with G incorporated opposite 1,*N*^2^-ε-G (and no frameshifts), in contrast to the results shown in Table S6. The only major differences in the earlier paper (32) were that the (longer) hpol η was expressed in a baculovirus-based system (instead of *E. coli*) and that a less sensitive mass spectrometer was used (Thermo DecaXP). In the earlier paper, a slippage mechanism was included that could have accounted for G insertion in the product (32). In reviewing the results of our present study, it is clear that both G and A were present in the extended primer product (Table 1 and Table S6, Fig. 6, Figs. S9, S11, S13, S14, S17 and S18), as evidenced by the presence of both in the products generated by hpol η from distinct oligonucleotides. A slippage mechanism is not very likely to explain the incorporation of A.

A result observed in the present work with oligonucleotide **2** is the insertion of A followed by misinsertion of G, apparently opposite the T immediately 5′ to the 1,*N*^2^-ε-G adduct (Table S6). A slippage mechanism would require insertion of A followed by a 2-base loop-out to the 5′ penultimate C and then realignment to complete the polymerization. Another anomaly was the apparent misincorporation of G opposite the 5′ T in about one-half of the product (but not with the unmodified oligonucleotide) (Table S6).

Presently, there are no crystal structures of ternary complexes with hpol η trapped at the extension step after 1,*N*^2^-ε-G bypass. The insertion stage complexes with either incoming dAMPnPP (Fig. 7) or dCMPnPP (Fig. 8) both reveal the adduct in the *syn* orientation. The absence of frameshifting in the full-length extension reactions supports the notion that the adduct maintains the *syn* orientation following TLS by hpol η. A coplanar arrangement of either G or A paired to 1,*N*^2^-ε-G at the −1 position can be expected to result in a locally distorted duplex geometry in order to accommodate the adducted and inserted purine moieties. Although extensions can proceed, they do so in an error-prone way. What is clear, however, is that an arrangement with the adduct in the *anti* orientation and either incoming dGTP or dATP stacked on top of 1,*N*^2^-ε-G during bypass is neither supported by the structural data nor by the sequences identified from the extension assays.

Our observations of hpol η-catalyzed TLS past the 1,*N*^2^-ε-G lesion reveal similarities in terms of the preferential insertion of purine nucleoside triphosphates by this pol opposite an array of other lesions. However, they also demonstrate contrasting underlying bypass mechanisms as well as the distinct means of hpol η to accommodate similar adducts, *e.g.*, the exocyclic 1,*N*^2^-ε-G and 1,*N*^6^-ε-A, at its active site. For example, with abasic sites the purine rule applies—hpol η rarely inserts T and C (33)—and A and G are also preferred for insertion over T opposite 1,*N*^6^-ε-A by hpol η (34). However, crystal structures of ternary complexes showed that 1,*N*^6^-ε-A is in the *anti* conformation when the preferred A and G are inserted. Also, distinct from the bypass of 1,*N*^2^-ε-G, the bypass of 1,*N*^6^-ε-A by hpol η involves frameshifts. Another distinct feature of the bypass reactions opposite the two exocyclic etheno adducts is that structural data showed that 1,*N*^6^-ε-A is in the *syn* conformation opposite dTTP (34). Conversely, we demonstrated here that 1,*N*^2^-ε-G is in the *syn* orientation both with incoming dAMPnPP and dCMPnPP. The origins of the different behaviors of the two adducts in terms of adopting either the *syn* or *anti* orientation at the active site of hpol η are currently not clear.

Along with shared and distinct features of the bypass of similar adducts by hpol η, it is also worth noting in the context of 1,*N*^2^-ε-G that *S. solfataricus* Dpo4 and hpol η, two Y-family pols that have much in common in terms of both activity and structure, behave very differently vis-à-vis this adduct. Thus, both pols have active sites with high ceilings that allow accommodation of two template bases and efficient bypass of CPDs and cisplatin intrastrand cross-links. However, bypass reactions opposite 1,*N*^2^-ε-G by Dpo4 involve the adduct in the *anti* orientation with a stacked incoming nucleotide that result in −1 frameshifts (31). These differences contrast sharply with our previous finding that both Dpo4 and hpol η are capable of mostly correct bypass of 8-oxoG (42, 46). In the case of Dpo4, the preference of 8-oxoG in the *anti* conformation opposite incoming dCTP *versus* in the *syn* conformation opposite incoming dATP is about 19:1. For hpol η the discrimination is even larger, with the percentage of insertion of dATP (and therefore 8-oxoG in *syn*) only between 1 and 3%. It is therefore remarkable that the bypass of 1,*N*^2^-ε-G is so different for the two pols, *i.e.*, in Dpo4, the adduct is always in the *anti* conformation but in hpol η it seems to be always in the *syn* conformation as per the two new structures.

## Experimental procedures

### hPol η catalytic core protein expression and purification

The hpol η plasmid (pET28a) comprising residues 1 to 432 was a generous gift from Dr. Wei Yang, National Institute of Diabetes and Digestive and Kidney Diseases, National Institutes of Health. The polymerase was expressed in *E. coli* and purified as described previously (47), and the protein solution was concentrated to ∼5 mg/ml.

### Materials

The 1,*N*^2^-ε-G-modified phosphoramidite (Fig. S1) was synthesized using a previously reported procedure (48). Unlabeled dNTPs and uracil DNA glycosylase (UDG) were purchased from New England Biolabs. C_18_ Sep-Pak columns were purchased from Waters. Piperidine was from Sigma-Aldrich. Unmodified oligonucleotides and FAM-labeled oligonucleotide primers were purchased from Integrated DNA Technologies. dAMPnPP and dCMPnPP were obtained from Jena Bioscience.

### 1,*N*^2^-ε-G-oligonucleotide synthesis, purification, and characterization

Solid-phase synthesis of the 1,*N*^2^-ε-G-modified oligonucleotides was done on a PerSeptive Biosystems Model 8909 DNA synthesizer (see Table S1 for in-house synthesis of modified oligonucleotide sequences) (Figs. S2 and S3). The modified DNAs were synthesized on a 1-μmol scale using the appropriate controlled pore glass (CPG) as solid support. Manual coupling for the modified phosphoramidite was carried out for 30 min. The modified oligonucleotides were deprotected using a reported procedure (49). The modified oligonucleotides were purified by denaturing polyacrylamide gel electrophoresis (20% PAGE, 7 M urea) at 55 W for 2.5 h using 1X TBE buffer (89 mM Tris-HCl buffer (pH 8.0) containing 89 mM boric acid and 2 mM EDTA). The gel was visualized under a UV lamp at 260 nm, and the desired oligonucleotide bands were isolated from the gel and extracted using TEN buffer (10 mM Tris-HCl buffer (pH 8.0) containing 1 mM EDTA and 300 mM NaCl) overnight at room temperature. The modified oligonucleotides were desalted using C_18_ Sep-Pak columns, and their integrity was confirmed by MALDI-TOF mass spectrometry (positive linear mode, Figs. S2 and S3).

### Full-length extension assays

A primer–template complex consisting of a FAM-labeled oligonucleotide primer and an unmodified or modified template was annealed (1:1 M ratio) at 95 °C for 5 min followed by slow cooling overnight (see Table S1 for oligonucleotide sequences) (Figure 3, Figure 4, Figure 5*A*–5*A* and Fig. S4*A*–S6*A*). Full-length extension reactions were carried out using 50 mM Tris-HCl buffer (pH 7.5) containing 50 mM NaCl, 5 mM MgCl_2_, 5% glycerol (v/v), 5 mM DTT, and 100 μg/ml BSA at 37 °C for 2 h. The final concentration of the primer–template complex was 150 nM, and 120 nM hpol η was used to obtain fully extended primers. Reactions were initiated by adding a 1.25 μl mixture of dNTPs to a total volume of 25 μl. Aliquots (3.5 μl) of reaction mixtures were removed at times of 0, 5, 30, 60, and 120 min and quenched with 6.5 μl of 10 mM EDTA (pH 8.0) in 95% deionized formamide (v/v). Products were separated using 20% PAGE (7 M urea). Results were visualized on a Typhoon scanner (GE Healthcare) and analyzed with ImageJ software.

### Single-nucleotide incorporation assays

Each primer–template oligonucleotide complex containing a FAM-labeled oligonucleotide primer and an unmodified or modified template was annealed (1:1 M ratio) at 95 °C for 5 min, followed by slow cooling overnight (see Table S1 for oligonucleotide sequences) (Figs. 3, *B* and *C*, 4, *B* and *C* and 5, *B* and *C*, Figs. S4, *B* and *C*, S5, *B* and *C*, and S6, *B* and *C*). All single-nucleotide insertion reactions were performed using 50 mM Tris-HCl buffer (pH 7.5) containing 50 mM NaCl, 5 mM MgCl_2_, 5% glycerol (v/v), 5 mM DTT, and 100 μg/ml BSA, at 37 °C. The final concentration of primer–template complex was 150 nM and 10 nM hpol η was used, followed by the addition of 1.25 μl of an individual dNTP to a total volume of 25 μl. Aliquots (3.5 μl) of reaction mixtures were removed at 0, 5, 10, and 30 min and quenched with 6.5 μl of 10 mM EDTA (pH 8.0) in 95% deionized formamide (v/v). Products were separated using 20% PAGE (7 M urea). Results were visualized on a Typhoon scanner (GE Healthcare) and analyzed with ImageJ software.

### Full-length extension reactions followed by UDG and piperidine treatment for LC-ESI-MS/MS analysis

A dU-containing FAM-labeled primer and an unmodified or modified template were annealed (1:1 M ratio) at 95 °C for 5 min followed by slow cooling overnight (see Table S1 for oligonucleotide sequences) (Fig. S7). The full-length extension reactions were carried out under similar reaction conditions as described in full-length extension assays, except that the final concentrations were as follows: the primer–template complex concentration was 2.5 μM and hpol η was 1.35 μM for the oligonucleotide 1,*N*^2^-ε-G template–primer complex and 0.75 μM for the unmodified template–primer complex, in a total reaction volume of 85 μl. Reactions were started using a mixture of dNTPs (1 mM) at 37 °C for 4 h. The resulting products were desalted using Biospin columns. The fully extended products were treated with 25 U of UDG at 37 °C for 4 h, followed by 0.25 M piperidine at 95 °C for 1 h (31, 35), and the reaction mixture was evaporated to dryness by lyophilization. The dried pellet was resuspended in 500 μl of nuclease-free water and concentrated to dryness by lyophilization. The samples were dissolved in 30 μl of nuclease-free water for LC-ESI-MS/MS analysis.

### LC-ESI-MS/MS analysis

LC-MS/MS analyses were performed on a Waters Acquity UPLC system connected directly to a Thermo-Finnigan LTQ mass spectrometer (electrospray ionization) (Fig. 6, Figs. S8–S19 and Tables S3–S10). The analysis was carried out in the negative ion mode using an octadecylsilane (C_18_) column (Acquity UPLC BEH, 1.7 μm, 2.1 mm × 100 mm) as reported previously (37). The extended product sequences were identified (Fig. 6, Figs. S8–S19, Table 1, Tables S3–S10) by comparing the theoretical *m/z* values using a Mongo Oligo Mass Calculator v2.06 with the observed CID fragments. For the coeluted products, relative peak areas were calculated on the basis of the intensity of representative fragments (as shown in Fig. S10 for a_5_-B(−2) and Figs. S15 and S19 for a_4_-B(−1) fragments). The relative yields of extended product sequences were calculated on the basis of relative peak areas of extracted ion chromatograms.

### Crystallization of complexes

Primer and template sequences used in the crystallization experiments are shown in Table S2 (Figs. 7 and 8, Figs. S20 and S21). The DNA template–primer duplex was mixed with the protein in a 1.2:1 M ratio in the presence of excess 50 mM Tris-HCl buffer (pH 7.5) containing 450 mM KCl and 3 mM DTT. After adding 5 microl of 100 mM MgCl_2_, the complex was concentrated to a final concentration of 2 to 3 mg/ml by ultrafiltration. Nonhydrolyzable nucleotide triphosphates were added last to form the ternary complexes. Crystallization experiments were performed by the hanging drop vapor diffusion technique at 18 °C using a sparse matrix screen (Hampton Research) (50). One microliter of the complex solution was mixed with 1 μl of reservoir solution and equilibrated against 500 μl reservoir wells. Crystals appeared in droplets containing 0.1 M MES buffer (pH 5.5) containing 5 mM MgCl_2_ and 21 to 23% (w/v) PEG 2000 MME within 1 day and were harvested after 1 week. Crystals were mounted in nylon loops, cryo-protected in reservoir solution containing 22% glycerol (v/v), and frozen in liquid nitrogen.

### X-ray diffraction data collection, structure determination, and refinement

Diffraction data were collected on the 21-ID-G beamline of the Life Sciences Collaborative Access Team (LS-CAT) at the Advanced Photon Source, Argonne National Laboratory. All data were integrated and scaled with the program HKL2000 (51). The structures were determined by the molecular replacement technique with the program MOLREP (52, 53), using the protein portion of structure of the hpol η complex with PDB ID 4O3N (42) as the search model. Structure refinement and model building were carried out with the programs Refmac (54) and COOT (55), respectively. Illustrations were prepared with the program UCSF Chimera (56). Final refinement parameters are summarized in Table 2. The quality of the model of the dAMPnPP complex structure is somewhat inferior compared with that of the dCMPnPP complex structure, in terms of both resolution and refinement parameters. However, we are confident that this structure too provides valid insights into the mechanism of hpol η-catalyzed bypass of the 1,*N*^2^-ε-G lesion.

## Data availability

With the exception of the Protein Data Bank coordinates (PDB 5F9I, 5F9N), all data is contained within the article and Supporting Information. The atomic coordinates and structure factors have been deposited in the Protein Data Bank (http://wwpdb.org/): PDB ID codes 5F9I (insertion 1,*N*^2^-ε-G opposite dAMPnPP) and 5F9N (insertion 1,*N*^2^-ε-G opposite dCMPnPP).

## Supporting information

This article contains supporting information.

## Conflict of interest

The authors declare that they have no conflicts of interest with the contents of this article.
