# Supplementary material for: Enzymatic bypass and the structural basis of miscoding opposite the DNA adduct 1,N2-ethenodeoxyguanosine by human DNA translesion polymerase η
Source: J Biol Chem. 2021 Apr 8;296:100642. doi: 10.1016/j.jbc.2021.100642 (PMC8121704; doi:10.1016/j.jbc.2021.100642)
Supplement: Supplemental Figures S1–S21 and Table S1–S10 [file mmc1.docx]

**Supporting Information**

**Enzymatic bypass and the structural basis of miscoding opposite the DNA adduct 1,*N*^2^-ethenodeoxyguanosine by human DNA translesion polymerase** η

Pratibha P. Ghodke^1,2^, Jyotirling R. Mali^1,3^, Amritraj Patra^1,2^, Carmelo J. Rizzo^2,3,4^, F. Peter Guengerich^2,4^, and Martin Egli^2,4,&^

*^2^Department of Biochemistry, Vanderbilt University, School of Medicine, Nashville, Tennessee 37232-0146; ^3^Department of Chemistry, Vanderbilt University, College of Arts and Science, Nashville, TN 37235; ^4^Vanderbilt-Ingram Cancer Center, 1301 Medical Center Drive 1710, Nashville, TN 37232*

**Table of Contents**

| Table S1 | List of oligonucleotide sequences | Page S3 |
| --- | --- | --- |
| Table S2 | Oligonucleotide sequences and nucleotides used in crystallization experiments | Page S4 |
| Figure S1 | ^31^P NMR of 1,*N*^2^-ethenodeoxyguanosine (1,*N*^2^-ε-G) modified phosphoramidite | Page S5 |
| Figure S2 | MALDI spectrum of 1,*N*^2^-ε-G modified oligonucleotide 1 (5´-T(εG)A-3´) | Page S6 |
| Figure S3 | MALDI spectrum of 1,*N*^2^-ε-G modified oligonucleotide 2 (5´-T(εG)G-3´) | Page S7 |
| Figure S4 | hpol η-mediated bypass across from 1,*N*^2^-ε-G in template 2 (5´-T(εG)G-3´) | Page S8 |
| Figure S5 | hpol η-mediated post-lesion full-length and single nucleotide insertion assays using 1,*N*^2^-ε-G in template 2 (5´-T(εG)G-3´) and Primer_6 | Page S9 |
| Figure S6 | hpol η-mediated post-lesion full-length and single nucleotide insertion assays using 1,*N*^2^-ε-G in template 2 (5´-T(εG)G-3´) and Primer_7 | Page S10 |
| Figure S7 | PAGE of full-length extension assays of LC-MS/MS analysis employing hpol η and mixture of dNTPs | Page S11 |
| Figure S8 | Extracted ion chromatogram for *m/z* 836.64 (−3, *t*_R_ 4.34 min.), indicating a mixture of two extended products (co-eluted) reactions for 1,*N*^2^-ε-G modified template 1 (5´-T(εG)A-3´) and Primer_4 | Page S12 |
| Table S3 | Observed and theoretical CID fragments of *m/z* 836.64 (−3) extended product sequence 5′-pCAT**AG**TGA-3′ for 1,*N*^2^-ε-G modified template 1 (5´-T(εG)A-3´) and Primer_4 | Page S13 |
| Figure S9 | Extracted ion chromatogram and CID spectrum for *m/z* 836.64 (−3, *t*_R_ 4.34 min) that stands for extended product sequence 5′-pCAT**G**ATGA-3′ for template 1 (5´-T(εG)A-3´) and Primer_4. | Page S14 |
| Table S4 | Observed and theoretical CID fragments of *m/z* 836.64 (−3) extended product sequence 5′-pCAT**G**ATGA-3′ for 1,*N*^2^-ε-G modified template 1 (5´-T(εG)A-3´) and Primer_4 | Page S15 |
| Figure S10 | Extracted ion chromatogram and zoom region of CID spectrum of *m/z* 836.64 (−3), a representative a_5_-B(−2) fragment from each product | Page S16 |
| Figure S11 | Extracted ion chromatogram and CID spectrum for *m/z* 842.00 (−3, *t*_R_ 4.34 min) that stands for extended product sequence 5′-pCAT**GG**TGA-3´ for template 1 (5´-T(εG)A-3´) and Primer_4 | Page S17 |
| Table S5 | Observed and theoretical CID fragments of *m/z* 842.00 (−3) extended product sequence is 5′-pCAT**GG**TGA-3′ for 1,*N*^2^-ε-G modified template 1 (5´-T(εG)A-3´) and Primer_4 | Page S18 |
| Table S6 | Summary of products of extension of 1,*N*^2^-ε-G modified template 2 (5´-T(εG)G-3´) and Primer_8 by hpol η analyzed by LC-MS/MS | Page S19 |
| Figure S12 | Extracted ion chromatogram for *m/z* 836.64 (−3, *t*_R_ 4.33 min), indicating a mixture of two extended products (co-eluted) for 1,*N*^2^-ε-G modified template 2 (5´-T(εG)G-3´) and Primer_8 | Page S20 |
| Figure S13 | Extracted ion chromatogram and CID spectrum for *m/z* 836.64 (−3, *t*_R_ 4.33 min) that stands for extended product sequence 5′-pTC**A**ATGAG-3′ for template 2 (5´-T(εG)G-3´) and Primer_8 | Page S21 |
| Table S7 | Observed and theoretical CID fragments of *m/z* 836.64 (−3) extended product sequence is 5′-pTC**A**ATGAG-3′ for 1,*N*^2^-ε-G modified template 2 (5´-T(εG)G-3´) and Primer_8 | Page S22 |
| Figure S14 | Extracted ion chromatogram and CID spectrum for *m/z* 836.64 (−3, *t*_R_ 4.33 min) that stands for extended product sequence 5′-pTC**G**ATGAA-3´ for template 2 (5´-T(εG)G-3´) and Primer_8 | Page S23 |
| Table S8 | Observed and theoretical CID fragments of *m/z* 836.64 (−3) extended product sequence is 5′-pTC**G**ATGAA-3′ for template 2 (5´-T(εG)G-3´) and Primer_8 | Page S24 |
| Figure S15 | Extracted ion chromatogram and zoom region of CID spectrum of *m/z* 836.64 (−3), a representative a_4_-B(−1) fragment from each product | Page S25 |
| Figure S16 | Extracted ion chromatogram for *m/z* 842.00 (−3, *t*_R_ 4.31 min), indicating a mixture of two extended products (co-eluted) for 1,*N*^2^-ε-G modified template 2 (5´-T(εG)G-3´) and Primer_8 | Page S26 |
| Figure S17 | Extracted ion chromatogram and CID spectrum for *m/z* 842.00 (−3, *t*_R_ 4.31 min) that stands for extended product sequence 5′-pTC**AG**TG**G**A-3′ for 1,*N*^2^-ε-G modified template 2 (5´-T(εG)G-3´) and Primer_8 | Page S27 |
| Table S9 | Observed and theoretical CID fragments of *m/z* 842.00 (−3) extended product sequence is 5′-pTC**AG**TG**G**A-3′ for 1,*N*^2^-ε-G modified template 2 (5´-T(εG)G-3´) and Primer_8 | Page S28 |
| Figure S18 | Extracted ion chromatogram and CID spectrum for *m/z* 842.00 (−3, *t*_R_ 4.31 min) that stands for extended product sequence 5′-pTC**G**ATG**G**A-3′ for 1,*N*^2^-ε-G modified template 2 (5´-T(εG)G-3´) and Primer_8 | Page S29 |
| Table S10 | Observed and theoretical CID fragments of *m/z* 836.64 (−3) extended product sequence is 5′-pTC**G**ATG**G**A-3′ for 1,*N*^2^-ε-G modified template 2 (5´-T(εG)G-3´) and Primer_8 | Page S30 |
| Figure S19 | Extracted ion chromatogram and zoom region of CID spectrum of *m/z* 842.00 (−3), a representative a_4_-B(−1) fragment from each product | Page S31 |
| Figure S20 | Quality of the final electron density in the active site region of the ternary complex between hpol η:1,*N*^2^-ε-G-adducted template-primer duplex and dAMPnPP | Page S32 |
| Figure S21 | Quality of the final electron density in the active site region of the ternary complex between hpol η:1,*N*^2^-ε-G-adducted template-primer duplex and dCMPnPP | Page S33 |

**Table S1.** List of oligonucleotide sequences

| Code | Oligonucleotide sequence | Calculated  [M + H]^+^ | Observed  [M + H]^+^ |
| --- | --- | --- | --- |
| 5´-T(εG)A-3´ (**1**) | 5´–CAT(1,*N*^2^-ε-G)ATGACGCTTCCCCC–3´  In-house synthesis | 5422.5630 | 5420.4621 |
| Control template_1 | 5´–CATGATGACGCTTCCCCC–3´  (IDT) |  |  |
| Primer_1 | 5´–FAM-GGGGGAAGCGTCAT–3´  (IDT) |  |  |
| Primer_2 | 5´–FAM-GGGGGAAGCGTCAT**C**–3´  (IDT) |  |  |
| Primer_3 | 5´–FAM-GGGGGAAGCGTCAT**A**–3´  (IDT) |  |  |
| Primer_4_LC-MS/MS | 5´–FAM-GGGGGAAGCG**U**CAT–3´  (IDT) |  |  |
| 5´-T(εG)G-3´ (**2**) | 5´–TCAT(1,*N*^2^-ε-G)GAATCCTTCCCCC–3´  In-house synthesis | 5396.5490 | 5395.6456 |
| Control template_2 | 5´–TCATGGAATCCTTCCCCC–3´  (IDT) |  |  |
| Primer_5 | 5´–FAM-GGGGGAAGGATTC–3´  (IDT) |  |  |
| Primer_6 | 5´–FAM-GGGGGAAGGATTC**C**–3´  (IDT) |  |  |
| Primer_7 | 5´–FAM-GGGGGAAGGATTC**A**–3´  (IDT) |  |  |
| Primer_8_LC-MS/MS | 5´–FAM-GGGGGAAGGA**U**TC–3´  (IDT) |  |  |

**Table S2.** Oligonucleotide sequences and nucleotides used in crystallization experiments

| Structure name | DNA sequence | Incoming nucleotide |
| --- | --- | --- |
| 1,*N*^2^-ε-G:dAMPnPP (Ai) | 3´- TCG CAG TA(1,*N*^2^-ε-G) TAC -5´  5´- AGC GTC AT -3´ | dAMPnPP |
| 1,*N*^2^-ε-G:dCMPnPP (Ci) |  | dCMPnPP |


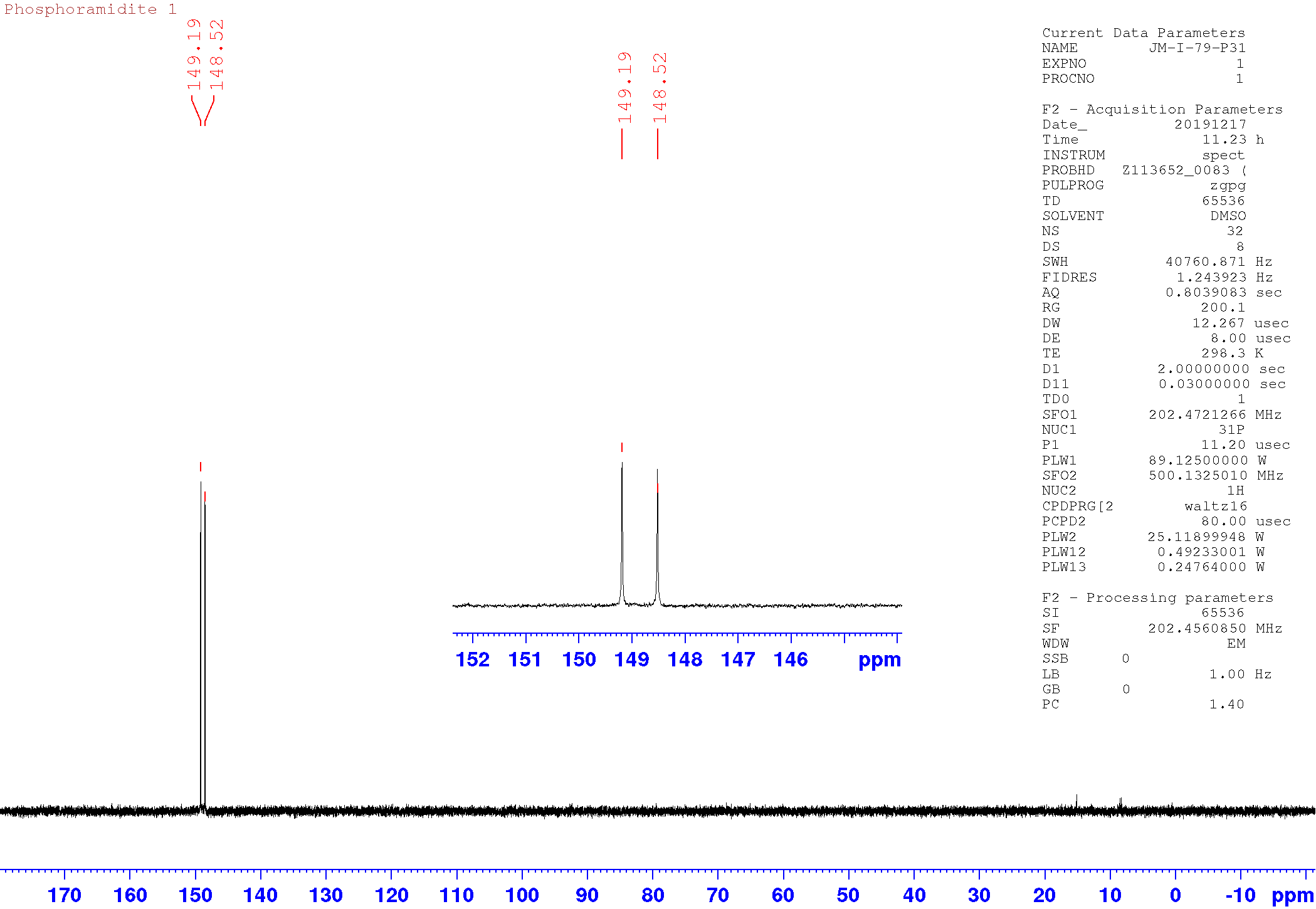


**Fig. S1.** ^31^P NMR of 1,*N*^2^-ethenodeoxyguanosine (1,*N*^2^-ε-G) modified phosphoramidite.


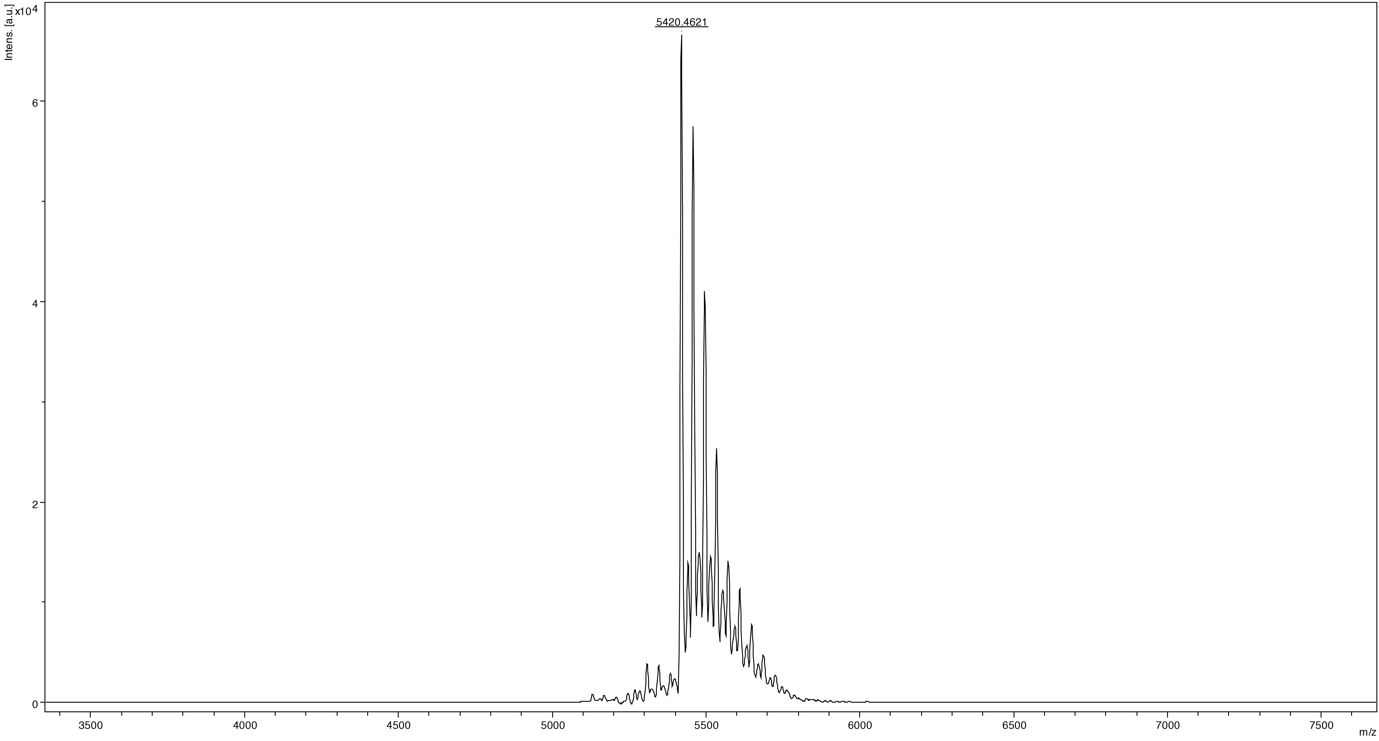


5´–CAT1,*N*^2^-ε-GATGACGCTTCCCCC–3´

**Fig. S2.** MALDI spectrum of 1,*N*^2^-ε-G-modified oligonucleotide 1 (5´-T(εG)A-3´).

Expected mass [M + H]^+^ 5422.5630; observed mass [M + H]^+^ 5420.4621.


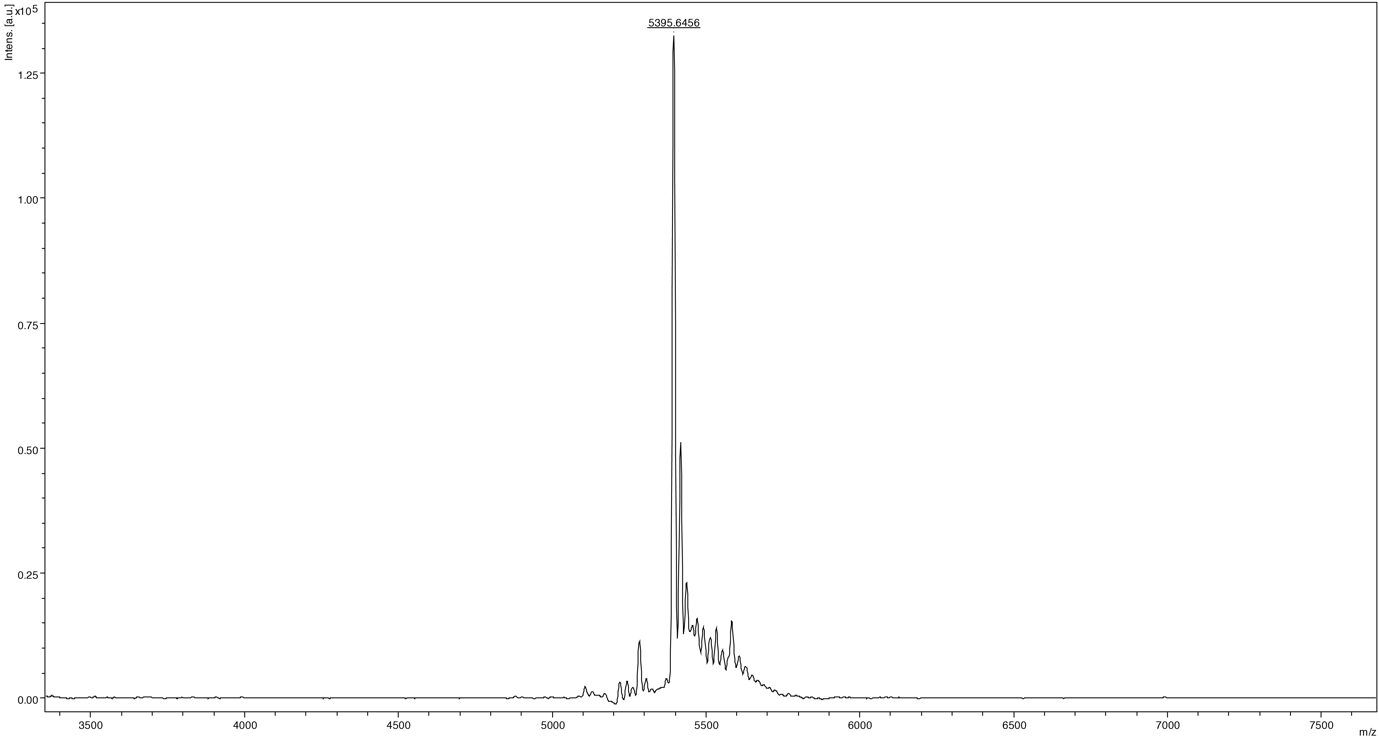


5´–TCAT1,*N*^2^-ε-GGAATCCTTCCCCC–3´

**Fig. S3.** MALDI spectrum of 1,*N*^2^-ε-G-modified oligonucleotide 2 (5´-T(εG)G-3´).

Expected mass [M + H]^+^ 5396.5490; observed mass [M + H]^+^ 5395.6456.


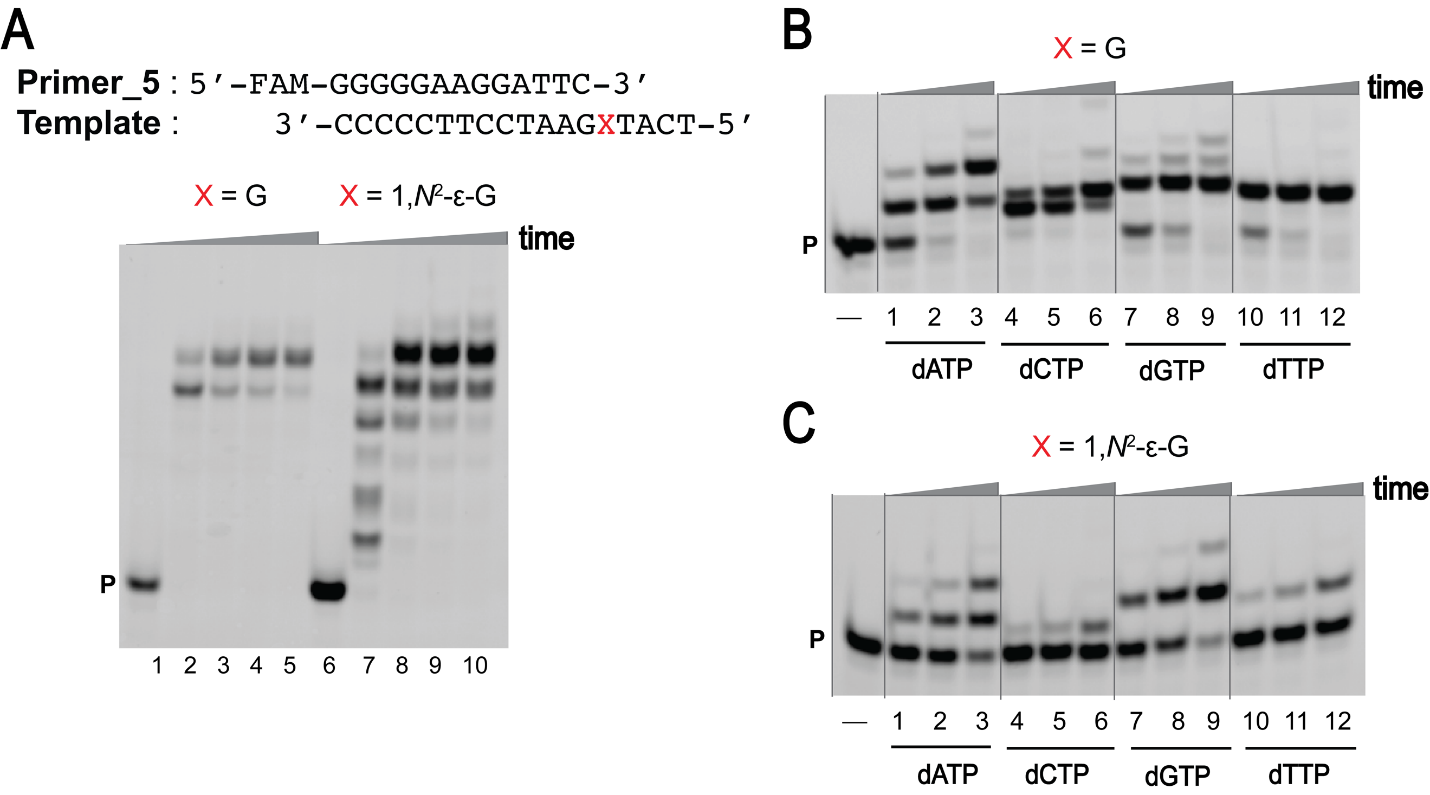


**Fig. S4.** **hpol η-mediated bypass across from 1,*N*^2^-ε-G in template 2 (5´-T(εG)G-3´).** PAGE (20%, 7 M urea): *A*, full-length extension assay: hpol η (120 nM) elongated Primer_5 opposite G- and 1,*N*^2^-ε-G-containing oligonucleotide templates in the presence of a mixture of dNTPs (500 μM). All reactions were done at 37 °C for 5-, 30-, 60-, and 120 min (time gradients indicated with wedges). Lanes: 1 to 5 for unmodified template; 6 to 10 for the 1,*N*^2^-ε-G-modified template. Single nucleotide insertion assays: hpol η (10 nM) was incubated with *B,* Primer_5/control template **2**, and *C*, Primer_5/1,*N*^2^-ε-G modified template **2** (5´-T(εG)G-3´) as well as with individual dNTPs (100 μM). Lanes: 1-3 for dATP, 4-6 for dCTP, 7-9 for dGTP, 10-12 for dTTP. All reactions were done at 37 °C for 5-, 10-, and 30 min. P indicates the FAM-labeled Primer_5. See Experimental Procedures and Table S1 for the oligonucleotide sequences used.


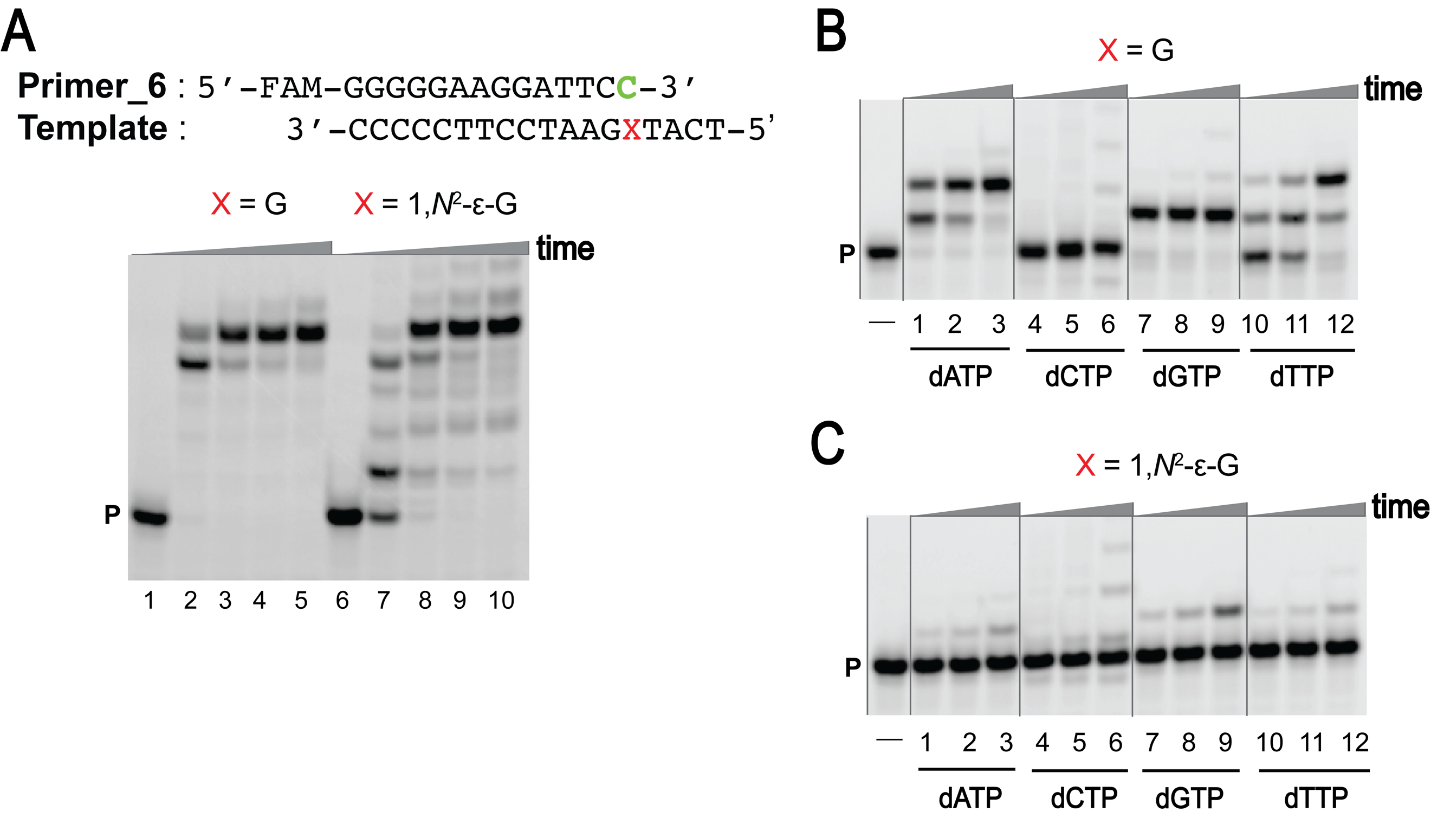


**Fig. S5.** **hpol η-mediated post-lesion full-length and single nucleotide insertion assays using 1,*N*^2^-ε-G in template 2 (5´-T(εG)G-3´) and Primer_6.** PAGE (20%, 7 M urea): *A*, Full-length extension assay: hpol η (120 nM) elongated oligonucleotide Primer_6 opposite G, and 1,*N*^2^-ε-G-containing oligonucleotide templates in the presence of a mixture of dNTPs (500 μM). All reactions were done at 37 °C for 5-, 30-, 60-, and 120 min (time gradients indicated with wedges). Lanes: 1 to 5 for unmodified template; 6 to 10 for 1,*N*^2^-ε-G-modified template. Single nucleotide insertion assays: hpol η (10 nM) was incubated with *B,* Primer_6/control template **2**, and *C*, Primer_6/1,*N*^2^-ε-G modified template **2** (5´-T(εG)G-3´) as well as with individual dNTPs (100 μM). Lanes: 1-3 for dATP, 4-6 for dCTP, 7-9 for dGTP, 10-12 for dTTP. All reactions were done at 37 °C for 5-, 10-, and 30 min. P indicates the FAM-labeled Primer_6. See Experimental Procedures and Table S1 for the oligonucleotide sequences used.


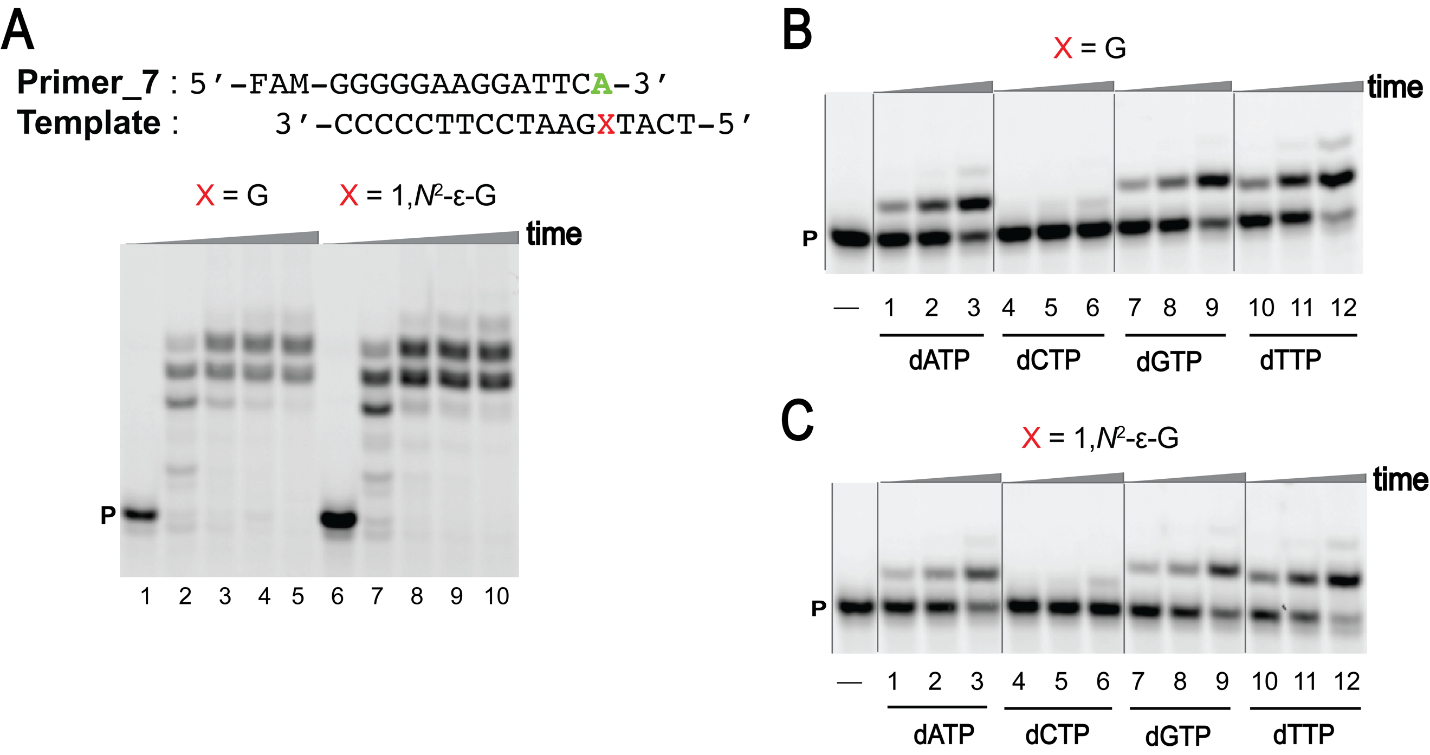


**Fig. S6.** **hpol η-mediated post-lesion full-length and single nucleotide insertion assays using 1,*N*^2^-ε-G in template 2 (5´-T(εG)G-3´) and Primer_7.** PAGE (20%, 7 M urea): *A*, full-length extension assay: hpol η (120 nM) elongated Primer_7 opposite G and 1,*N*^2^-ε-G-containing oligonucleotide templates in the presence of a mixture of dNTPs (500 μM). All reactions were done at 37 °C for 5-, 30-, 60-, and 120 min (time gradients indicated with wedges). Lanes: 1 to 5 for unmodified template; 6 to 10 for 1,*N*^2^-ε-G modified template. Single nucleotide insertion assays: hpol η (10 nM) was incubated with *B,* Primer_7/control template **2**, and *C*, Primer_7/1,*N*^2^-ε-G modified template **2** (5´-T(εG)G-3´) as well as with individual dNTPs (100 μM). Lanes: 1-3 for dATP, 4-6 for dCTP, 7-9 for dGTP, 10-12 for dTTP. All reactions were done at 37 °C for 5-, 10-, and 30 min. P indicates the FAM-labeled Primer_7. See Experimental Procedures and Table S1 for the oligonucleotide sequences used.


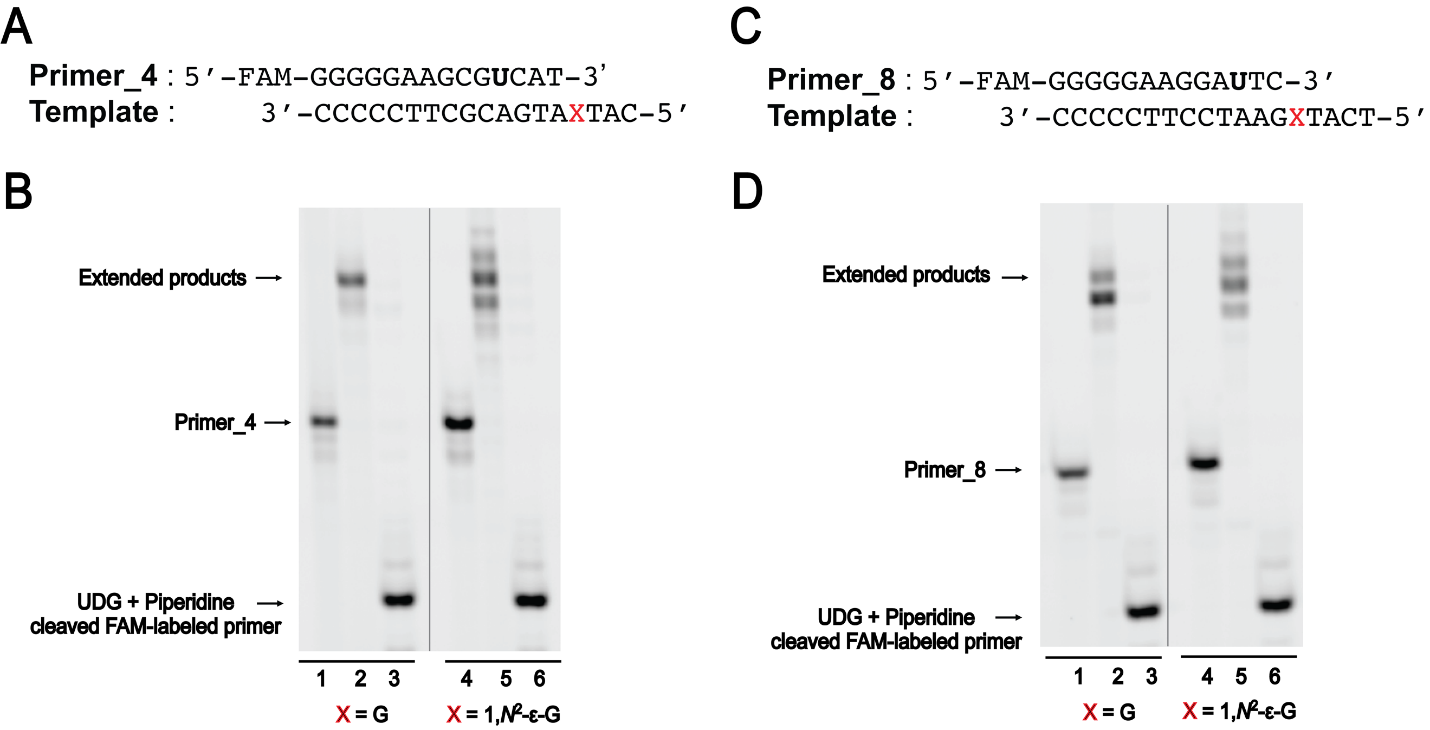


**Fig. S7. PAGE of full-length extension assays for LC-MS/MS analysis employing hpol η and mixture of dNTPs.** *A and C*, primer-template complexes; *B and D*, full-length extension reactions for unmodified as well as modified template **1** (5´-T(εG)A-3´)/Primer_4 and modified template **2** (5´-T(εG)G-3´)/Primer_8 complexes respectively (Lane 1 and 4 included FAM-labeled dU- containing primer, Lane 2 and 5 included fully-extended products, and Lanes 3 and 6 included cleaved FAM-labeled primer after UDG and piperidine treatment. All reactions were carried out at 37 °C for 4 h.


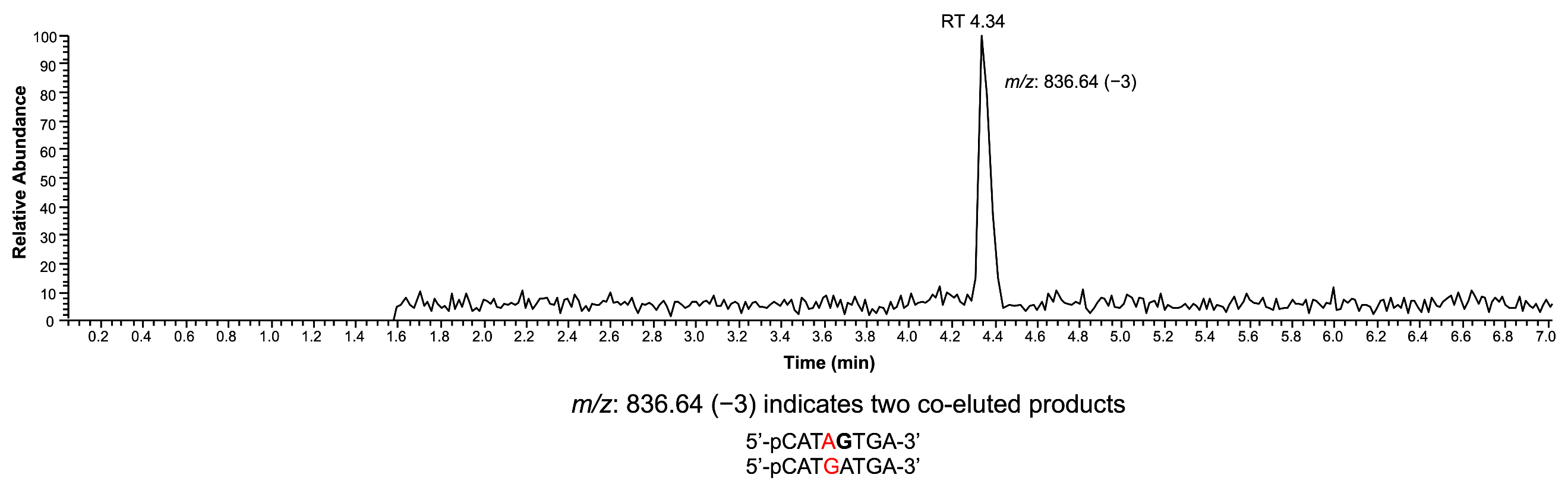


**Fig. S8.** **LC-MS/MS sequencing analysis of full-length extension reactions for 1,*N*^2^-ε-G modified** **template 1 (5´-T(εG)A-3´) and Primer_4.** Extracted ion chromatogram for *m/z* 836.64 (−3, *t*_R_ 4.34 min.), indicating a mixture of two extended products (co-eluted). The representative fragments for these products, a_5_-B(−2) are shown in Fig. S10. The fragmentation patterns for these products are presented in Figs. 6, S9 and Tables S3-S4.

**Table S3**

**Observed and theoretical CID fragments of *m/z* 836.64 (−3) from full-length extended products for 1,*N*^2^-ε-G modified** **template 1 (5´-T(εG)A-3´) and Primer_4**

The extended product sequence is 5′-pCAT**AG**TGA-3′ (Fig. 6), indicating insertion of A, followed by misinsertion of G plus blunt end addition of A.

| Fragment assignment | *m/z* observed | *m/z* theoretical |
| --- | --- | --- |
| 5′-pCATA (a_4_-B, −1) | 1083.27 | 1083.66 |
| 5′-pCATAG (a_5_-B, −2) | 698.00 | 697.93 |
| 5′-pCATAGTG (a_7_-B, −3) | 678.18 | 676.09 |
| pATAGTGA-3′ (W_7_, −3) | 738.73 | 740.47 |
| pTAGTGA-3′ (W_6_, −2) | 954.00 | 954.61 |
| pGTGA-3′ (W_4_, −1) | 646.27 | 645.91 |
| pTGA-3′ (W_3_, −1) | 963.18 | 963.62 |
| pA-3′ (W_1_, −1) | 330.00 | 330.21 |


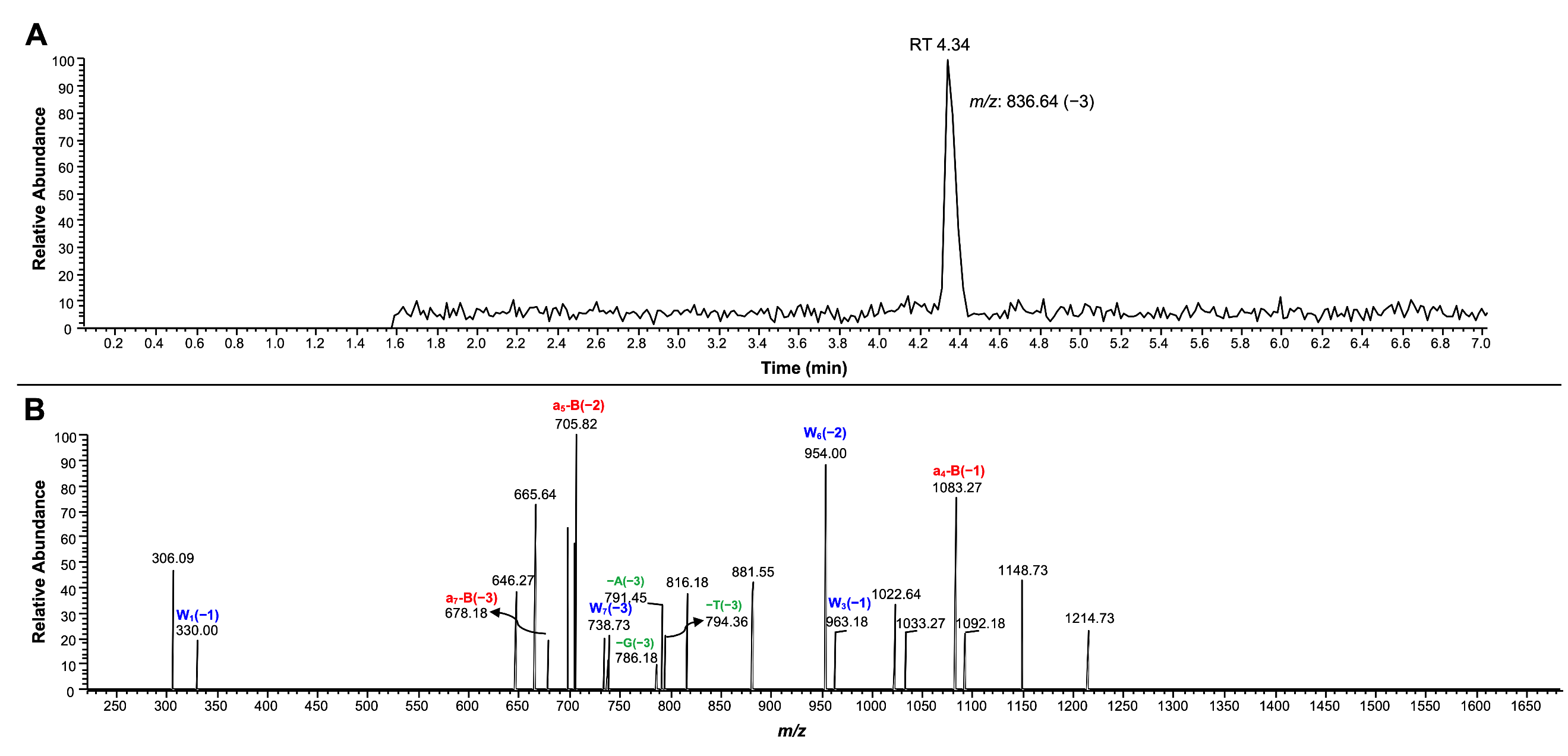


**Fig. S9. LC-MS/MS sequencing analysis of full-length extension reactions for template 1 (5´-T(εG)A-3´) and Primer_4.** *A*, extracted ion chromatogram for *m/z* 836.64 (−3, *t*_R_ 4.34 min) associated with the extended product sequence 5′-pCAT**G**ATGA-3′; *B*, CID spectrum of *m/z* 836.64 (−3). See Table S4 for fragment assignment.

**Table S4**

**Observed and theoretical CID fragments of *m/z* 836.64 (−3) from full-length extended products for 1,*N*^2^-ε-G modified** **template 1 (5´-T(εG)A-3´) and Primer_4**

The extended product sequence is 5′-pCAT**G**ATGA-3′ (Fig. S9*B*), indicating insertion of G plus blunt end addition of A.

| Fragment assignment | *m/z* observed | *m/z* theoretical |
| --- | --- | --- |
| 5′-pCATG (a_4_-B, −1) | 1083.27 | 1083.66 |
| 5′-pCATGA (a_5_-B, −2) | 705.82 | 705.93 |
| 5′-pCATGATG (a_7_-B, −3) | 678.18 | 676.09 |
| pATGATGA-3′ (W_7_, −3) | 738.73 | 740.47 |
| pTGATGA-3′ (W_6_, −2) | 954.00 | 954.61 |
| pTGA-3′ (W_3_, −1) | 963.18 | 963.62 |
| pA-3′ (W_1_, −1) | 330.00 | 330.21 |


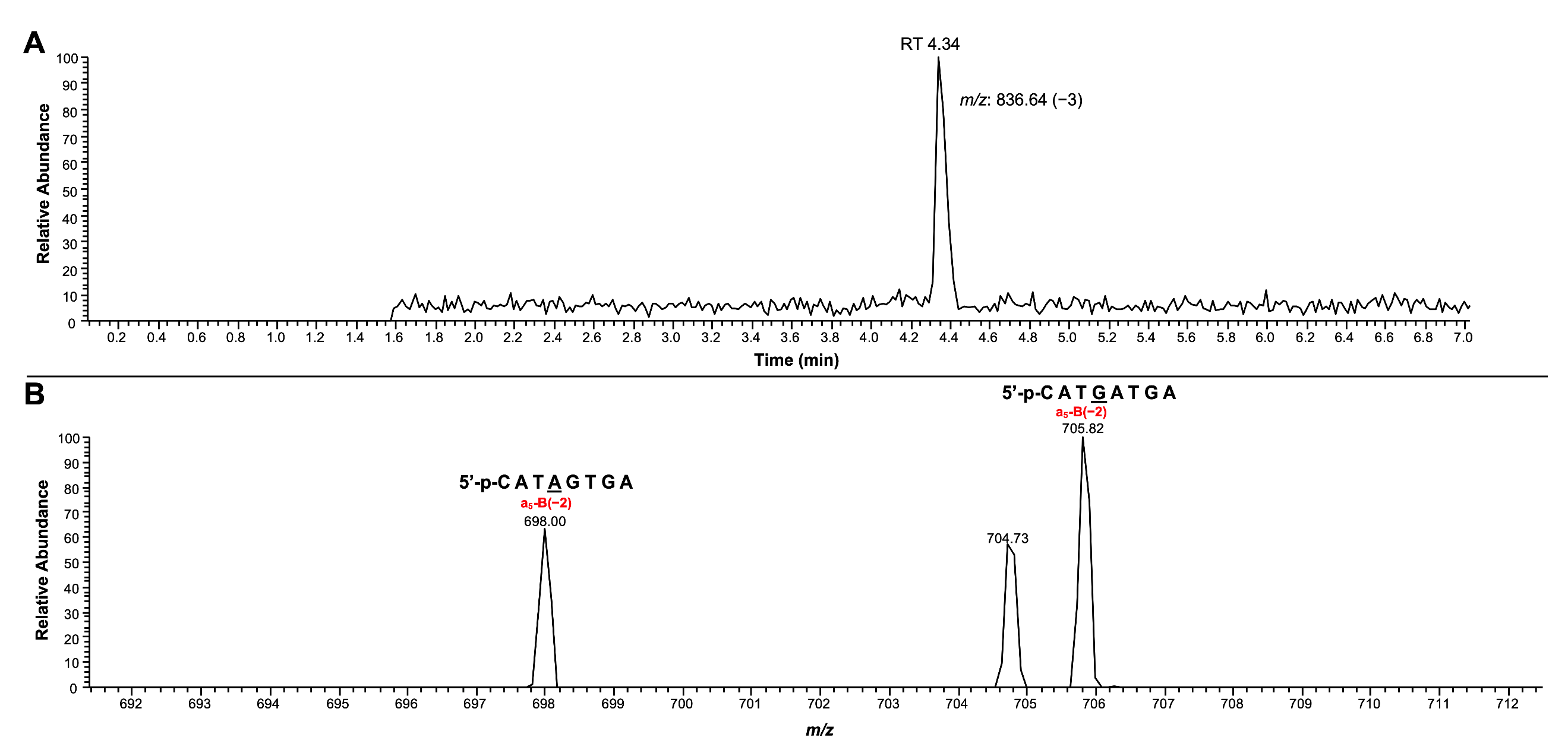


**Fig. S10. LC-MS/MS sequencing analysis of full-length extension reactions for template 1 (5´-T(εG)A-3´) and Primer_4.** *A*, extracted ion chromatogram for *m/z* 836.64 (−3, *t*_R_ 4.34 min); *B*, expanded region of CID spectrum of *m/z* 836.64 (−3). A representative a_5_-B(−2) fragment from each product is shown.


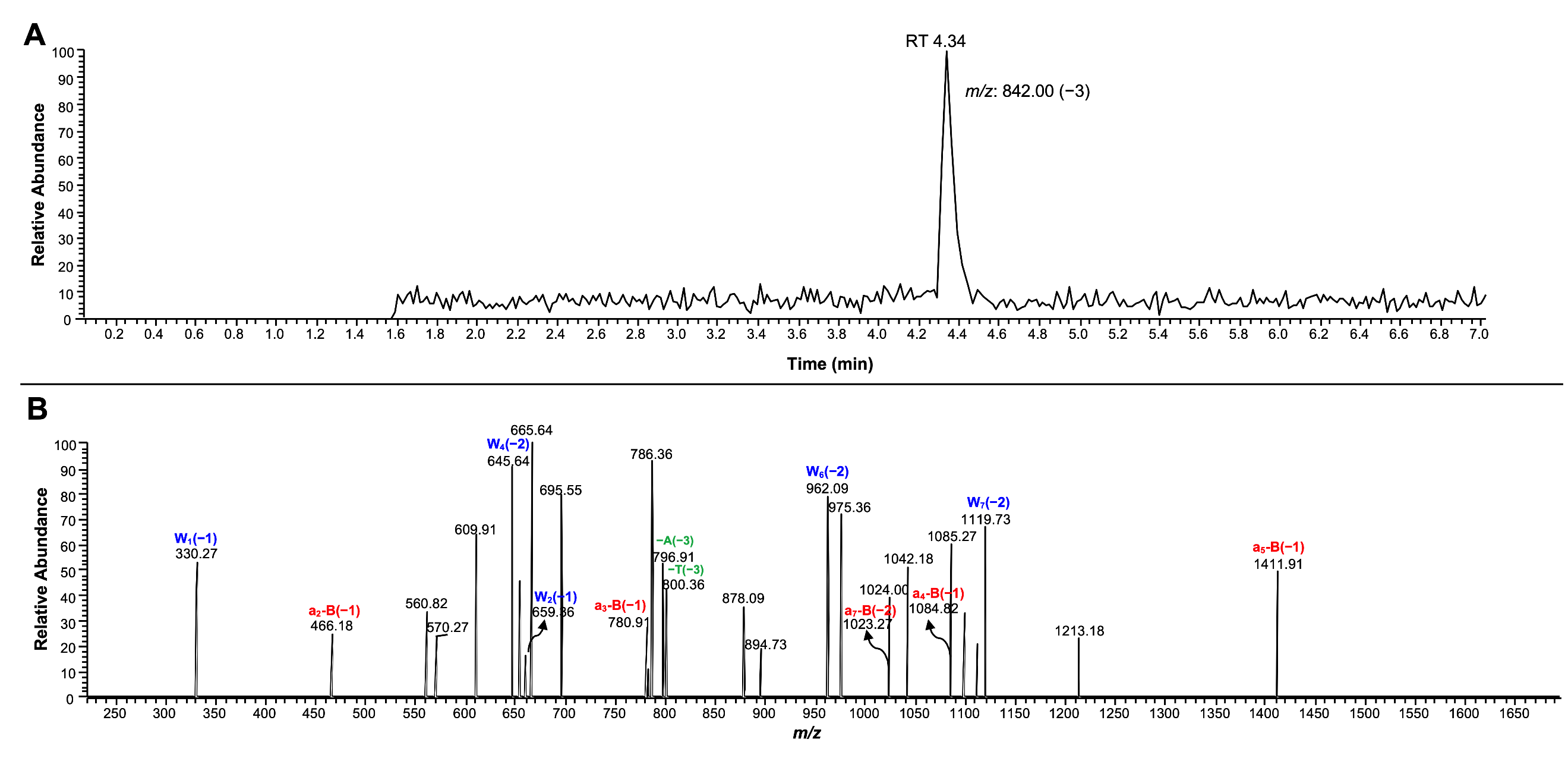


**Fig. S11. LC-MS/MS sequencing analysis of full-length extension reactions for template 1 (5´-T(εG)A-3´) and Primer_4.** *A*, extracted ion chromatogram for *m/z* 842.00 (−3, *t*_R_ 4.34 min) for the extended product sequence 5′-pCAT**GG**TGA-3′; *B*, CID spectrum of *m/z* 842.00 (−3). See Table S5 for fragment assignments.

**Table S5**

**Observed and theoretical CID fragments of *m/z* 842.00 (−3) from full-length extended products for 1,*N*^2^-ε-G modified** **template 1 (5´-T(εG)A-3´) and Primer_4**

The extended product sequence is 5′-pCAT**GG**TGA-3′ (Fig. S11*B*), indicating insertion of G, followed by misinsertion of G plus blunt end addition of A.

| **Fragment assignment** | ***m/z* observed** | ***m/z* theoretical** |
| --- | --- | --- |
| 5′-pCA (a_2_-B, −1) | 466.18 | 466.26 |
| 5′-pCAT (a_3_-B, −1) | 780.91 | 779.47 |
| 5′-pCATG (a_4_-B, −1) | 1084.82 | 1083.66 |
| 5′-pCATGG (a_5_-B, −2) | 1411.91 | 1412.87 |
| 5′-pCATGGTG (a_7_-B, −2) | 1023.27 | 1022.63 |
| pATGGTGA-3′ (W_7_, −2) | 1119.73 | 1119.22 |
| pTGGTGA-3′ (W_6_, −2) | 962.09 | 962.61 |
| pGTGA-3′ (W_4_, −1) | 645.64 | 645.91 |
| pGA-3′ (W_2_, −1) | 659.86 | 659.42 |
| pA-3′ (W_1_, −1) | 330.27 | 330.21 |

**Table S6**

**Summary of products of extension of 1,*N*^2^-ε-G modified** **template 2 (5´-T(εG)G-3´) and Primer_8 by hpol η analyzed by LC-ESI-MS/MS**

| Primer_8: 5´-FAM-GGGGGAAGGA**U**TC-3´  Template: 3´-CCCCCTTCCTAAG**X**TACT-5´ | | | | |
| --- | --- | --- | --- | --- |
| X | Sequence | Yield | Observed *m/z* | Base added |
| G | 5´-pTC**C**ATGAA-3´ | 60% | 823.55 (−3) | C, plus blunt end  addition of A and  G |
|  | 5´-pTC**C**ATGAG-3´ | 40% | 829.00 (−3) |  |
| 1,*N*^2^-ε-G | 5´-pTC**A**ATGAG-3´ | 20% | 836.64 (−3) | A, plus blunt end  addition of G |
|  | 5´-pTC**G**ATGAA-3´ | 25% | 836.64 (−3) | G, plus blunt end  addition of A |
|  | 5´-pTC**AG**TG**G**A-3´ | 26% | 842.00 (−3) | A, followed by  misinsertion of G,  misinsertion of G  at 5´T, plus, blunt  end addition of A |
|  | 5´-pTC**G**ATG**G**A-3´ | 29% | 842.00 (−3) | G, misinsertion of G  at 5´T, plus blunt  end addition of A |


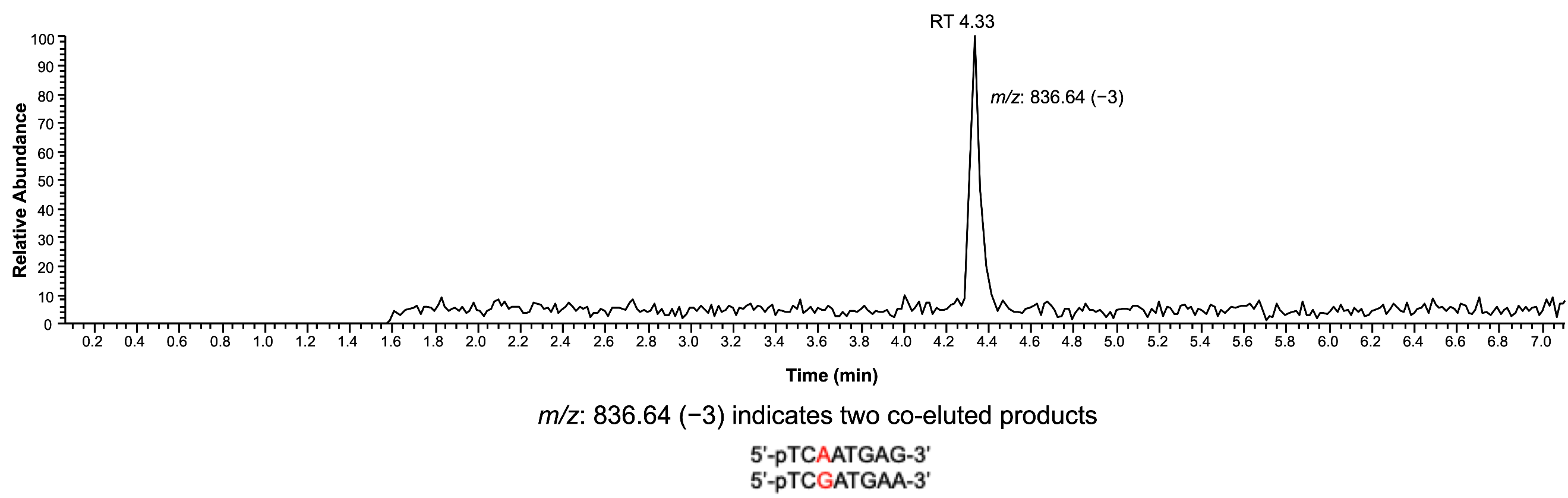


**Fig. S12.** **LC-MS/MS sequencing analysis of full-length extension reactions for 1,*N*^2^-ε-G modified** **template 2 (5´-T(εG)G-3´) and Primer_8.** Extracted ion chromatogram for *m/z* 836.64 (−3, *t*_R_ 4.33 min), indicating a mixture of two extended products (co-eluted). The fragments for these products, a_4_-B(−1), are shown in Fig. S15. The fragmentation patterns for these products are mentioned in Figs. S13-S14 and Tables S7-S8.


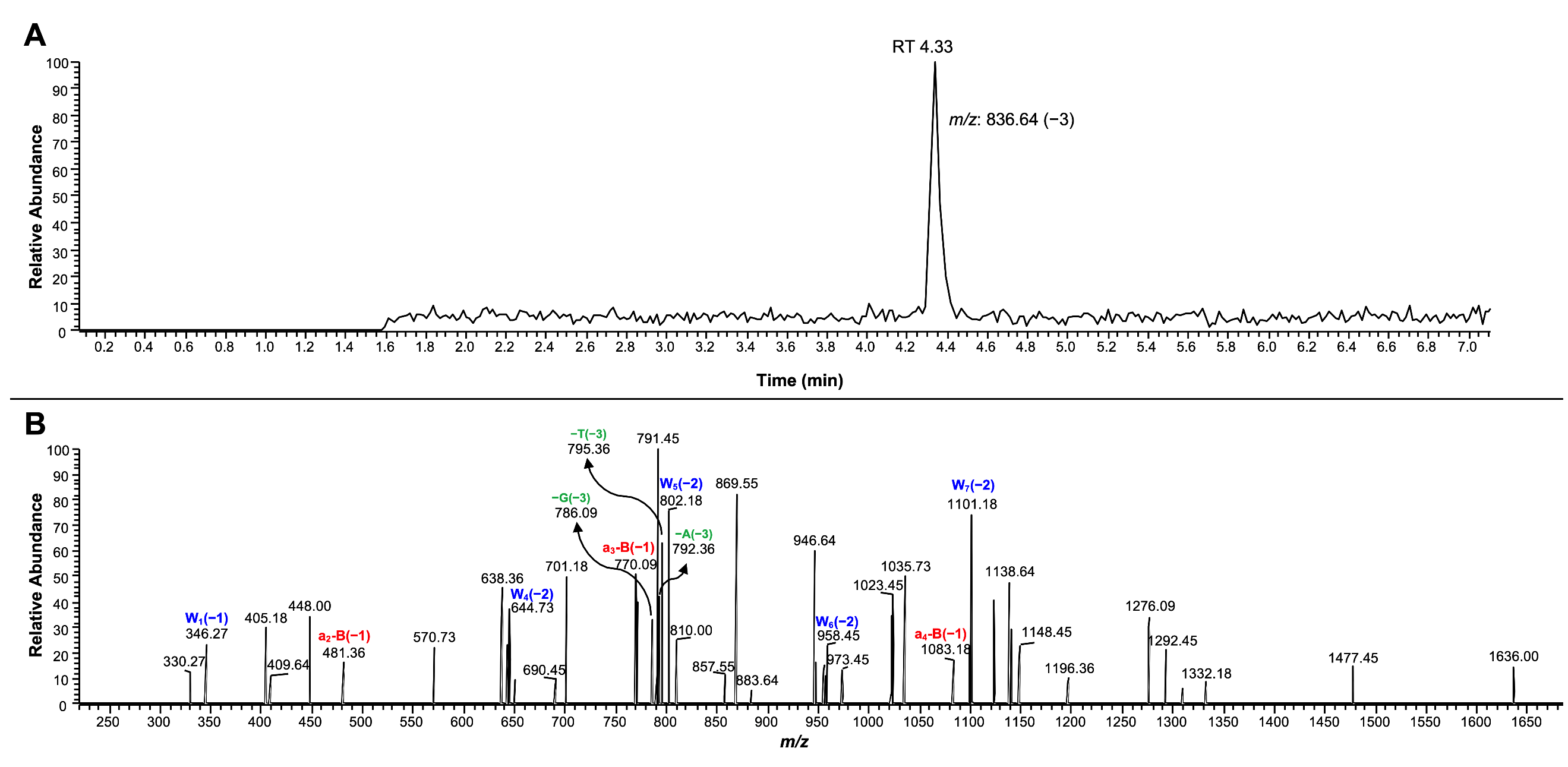


**Fig. S13. LC-MS/MS sequencing analysis of full-length extension reactions for template 2 (5´-T(εG)G-3´) and Primer_8.** *A*, extracted ion chromatogram for *m/z* 836.64 (−3, *t*_R_ 4.33 min) for extended product sequence 5′-pTC**A**ATGAG-3′; *B*, CID spectrum of *m/z* 836.64 (−3). See Table S7 for fragment assignment.

**Table S7**

**Observed and theoretical CID fragments of *m/z* 836.64 (−3) from full-length extended products for 1,*N*^2^-ε-G modified** **template 2 (5´-T(εG)G-3´) and Primer_8**

The extended product sequence is 5′-pTC**A**ATGAG-3′ (Fig. S13*B*), indicating insertion of A, plus blunt end addition of G.

| **Fragment assignment** | ***m/z* observed** | ***m/z* theoretical** |
| --- | --- | --- |
| 5′-pTC (a_2_-B, −1) | 481.36 | 481.27 |
| 5′-pTCA (a_3_-B, −1) | 770.09 | 770.45 |
| 5′-pTCAA (a_4_-B, −1) | 1083.18 | 1083.66 |
| pCAATGAG-3′ (W_7_, −2) | 1101.18 | 1103.71 |
| pAATGAG-3′ (W_6_, −2) | 958.45 | 959.12 |
| pATGAG-3′ (W_5_, −2) | 802.18 | 802.51 |
| pTGAG-3′ (W_4_, −1) | 644.73 | 645.91 |
| pG-3′ (W_1_, −1) | 346.27 | 346.21 |


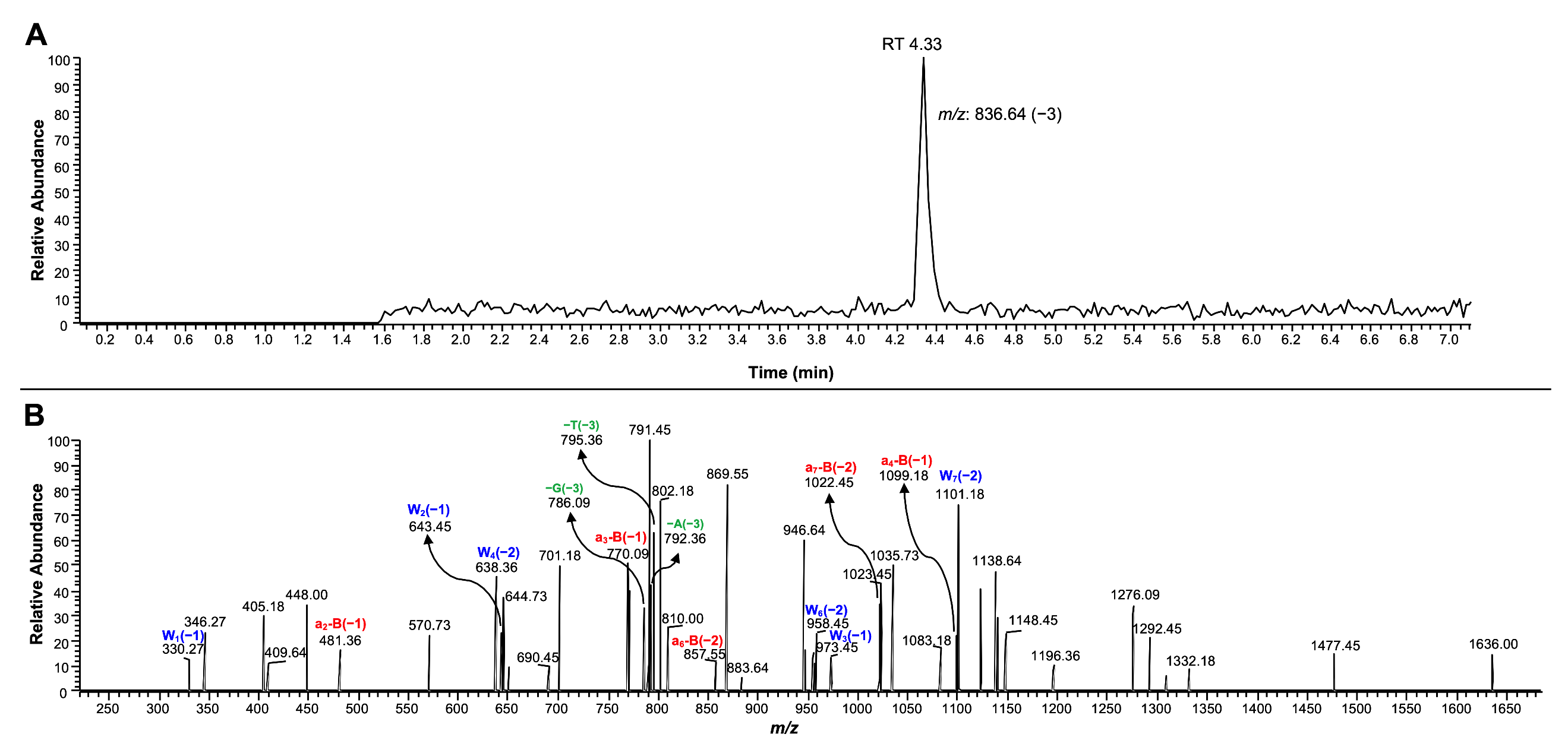


**Fig. S14. LC-MS/MS sequencing analysis of full-length extension reactions for template 2 (5´-T(εG)G-3´) and Primer_8.** *A*, extracted ion chromatogram for *m/z* 836.64 (−3, *t*_R_ 4.33 min) for extended product sequence 5′-pTC**G**ATGAA-3′; *B*, CID spectrum of *m/z* 836.64 (−3). See Table S8 for fragment assignment.

**Table S8**

**Observed and theoretical CID fragments of *m/z* 836.64 (−3) from full-length extended products for 1,*N*^2^-ε-G modified** **template 2 (5´-T(εG)G-3´) and Primer_8**

The extended product sequence is 5′-pTC**G**ATGAA-3′ (Fig. S14*B*), indicating insertion of G, plus blunt end addition of A.

| **Fragment assignment** | ***m/z* observed** | ***m/z* theoretical** |
| --- | --- | --- |
| 5′-pTC (a_2_-B, −1) | 481.36 | 481.27 |
| 5′-pTCG (a_3_-B, −1) | 770.09 | 770.45 |
| 5′-pTCGA (a_4_-B, −1) | 1099.18 | 1099.66 |
| 5′-pTCGATG (a_6_-B, −2) | 857.55 | 858.03 |
| 5′-pTCGATGA (a_7_-B, −2) | 1022.45 | 1022.63 |
| pCGATGAA-3′ (W_7_, −2) | 1101.18 | 1103.71 |
| pGATGAA-3′ (W_6_, −2) | 958.45 | 959.12 |
| pTGAA-3′ (W_4_, −1) | 638.36 | 637.91 |
| pGAA-3′ (W_3_, −1) | 973.45 | 972.63 |
| pAA-3′ (W_2_, −1) | 643.45 | 643.42 |
| pA-3′ (W_1_, −1) | 330.27 | 330.21 |


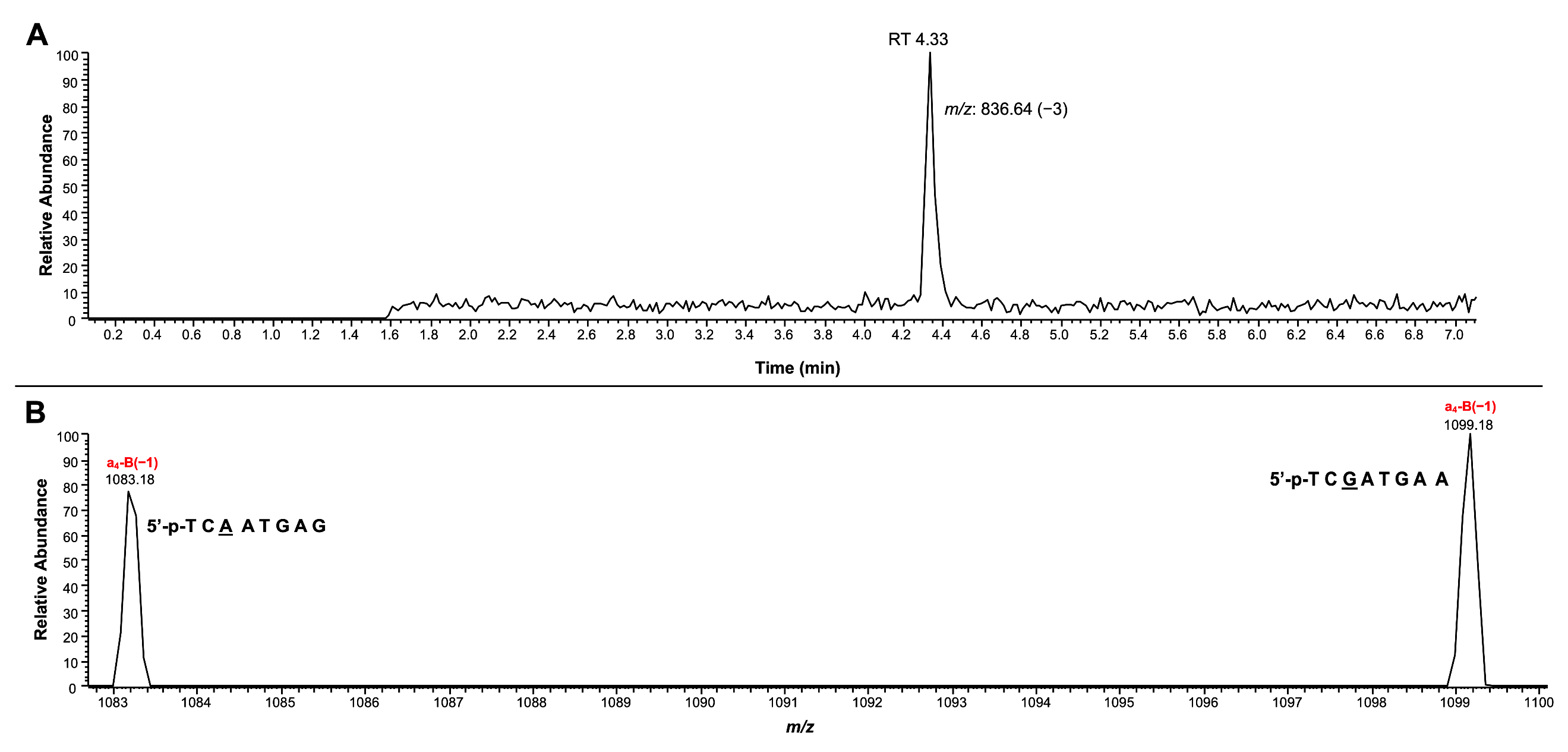


**Fig. S15. LC-MS/MS sequencing analysis of full-length extension reactions for template 2 (5´-T(εG)G-3´) and Primer_8.** *A*, extracted ion chromatogram for *m/z* 836.64 (−3, *t*_R_ 4.33 min); *B*, expanded region of CID spectrum of *m/z* 836.64 (−3), a representative a_4_-B(−1) fragment from each product is shown.


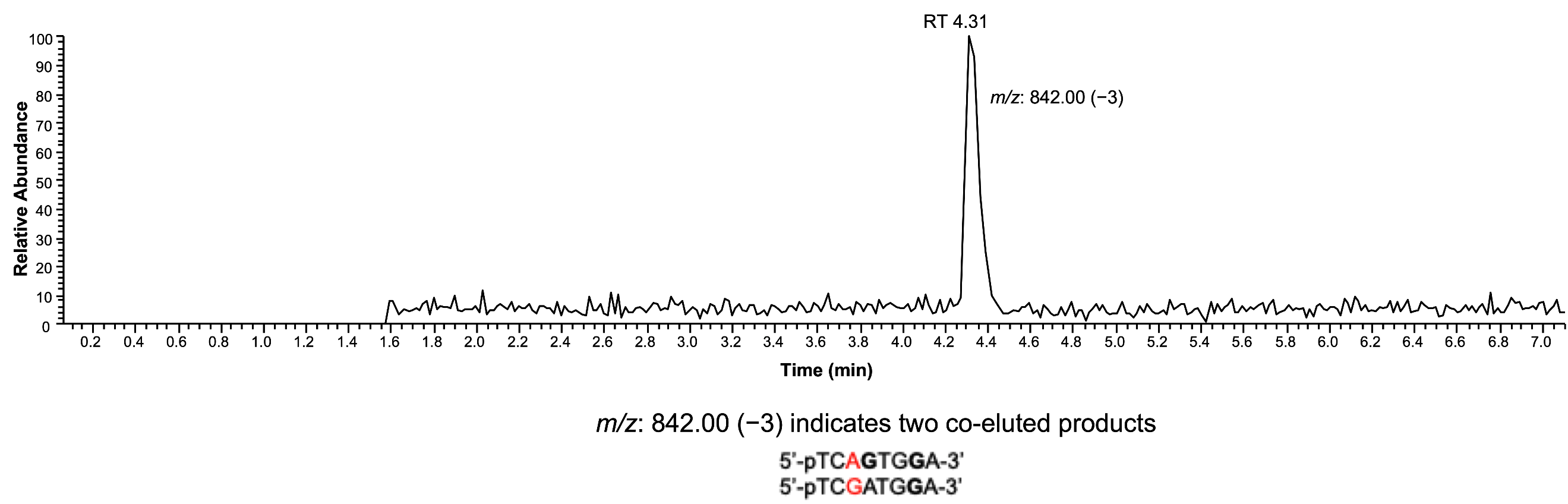


**Fig. S16.** **LC-ESI-MS/MS sequencing analysis of full-length extension reactions for 1,*N*^2^-ε-G modified** **template 2 (5´-T(εG)G-3´) and Primer_8.** Extracted ion chromatogram for *m/z* 842.00 (−3, *t*_R_ 4.31 min), indicating a mixture of two extended products (co-eluted). The representative fragments for these products, a_4_-B(−1) are shown in Fig. S19. The fragmentation patterns for these products are presented in Figs. S17-S18 and Tables S9-S10.


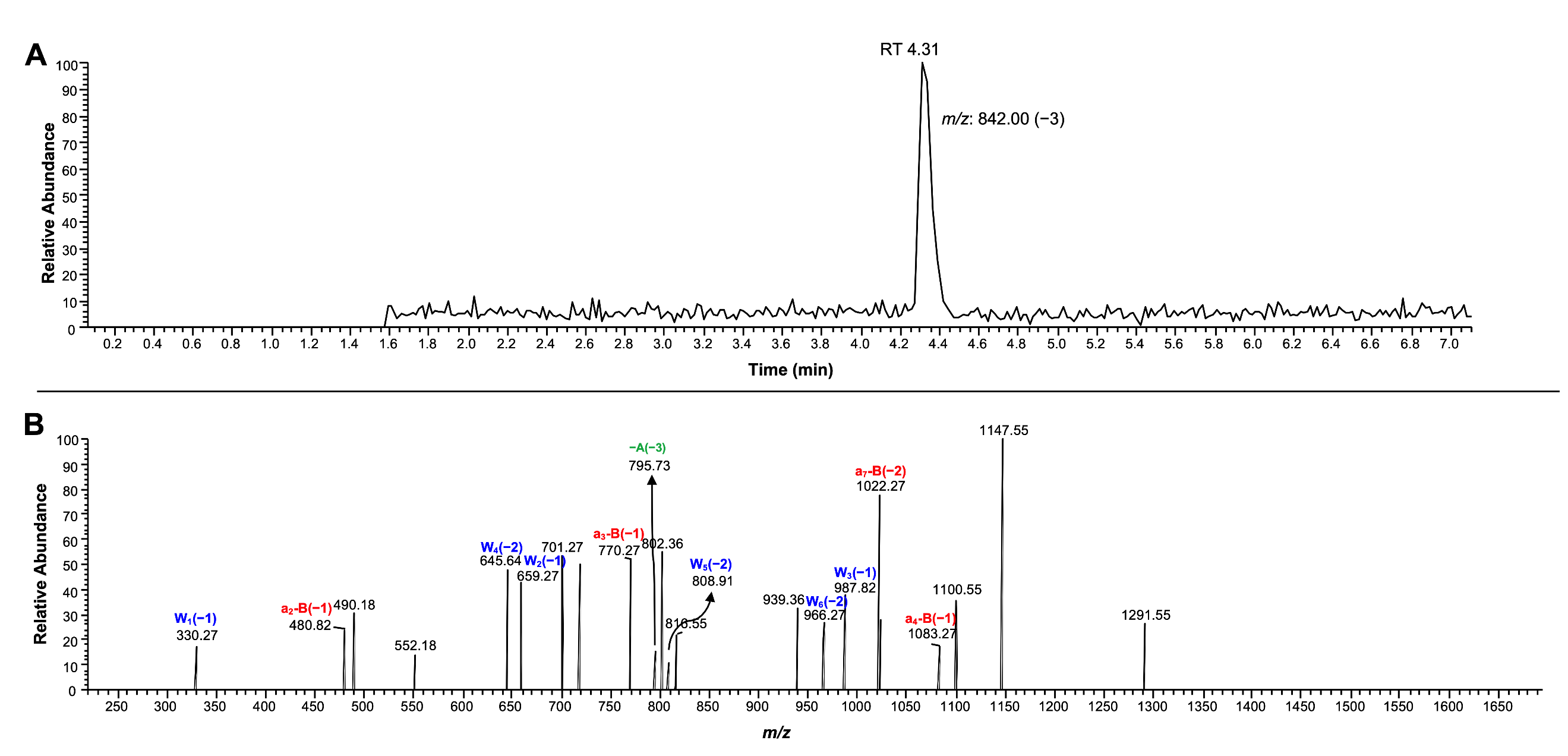


**Fig. S17. LC-MS/MS sequencing analysis of full-length extension reactions for 1,*N*^2^-ε-G modified** **template 2 (5´-T(εG)G-3´) and Primer_8.** *A*, extracted ion chromatogram for *m/z* 842.00 (−3, *t*_R_ 4.31 min) for extended product sequence 5′-pTC**AG**TG**G**A-3′; *B*, CID spectrum of *m/z* 842.00 (−3). See Table S9 for fragment assignment.

**Table S9**

**Observed and theoretical CID fragments of *m/z* 842.00 (−3) from full-length extended products for 1,*N*^2^-ε-G modified** **template 2 (5´-T(εG)G-3´) and Primer_8**

The extended product sequence is 5′-pTC**AG**TG**G**A-3′ (Fig. S17*B*), indicating insertion of A, followed by misinsertion of G, misinsertion of G at 5´T, plus, blunt end addition of A.

| **Fragment assignment** | ***m/z* observed** | ***m/z* theoretical** |
| --- | --- | --- |
| 5′-pTC (a_2_-B, −1) | 480.82 | 481.27 |
| 5′-pTCA (a_3_-B, −1) | 770.27 | 770.45 |
| 5′-pTCAG (a_4_-B, −1) | 1083.27 | 1083.66 |
| 5′-pTCAGTGG (a_7_-B, −2) | 1022.27 | 1022.63 |
| pAGTGGA-3′ (W_6_, −2) | 966.27 | 967.12 |
| pGTGGA-3′ (W_5_, −2) | 808.91 | 810.51 |
| pTGGA-3′ (W_4_, −1) | 645.64 | 645.91 |
| pGGA-3′ (W_3_, −1) | 987.82 | 988.63 |
| pGA-3′ (W_2_, −1) | 659.27 | 659.42 |
| pA-3′ (W_1_, −1) | 330.27 | 330.21 |


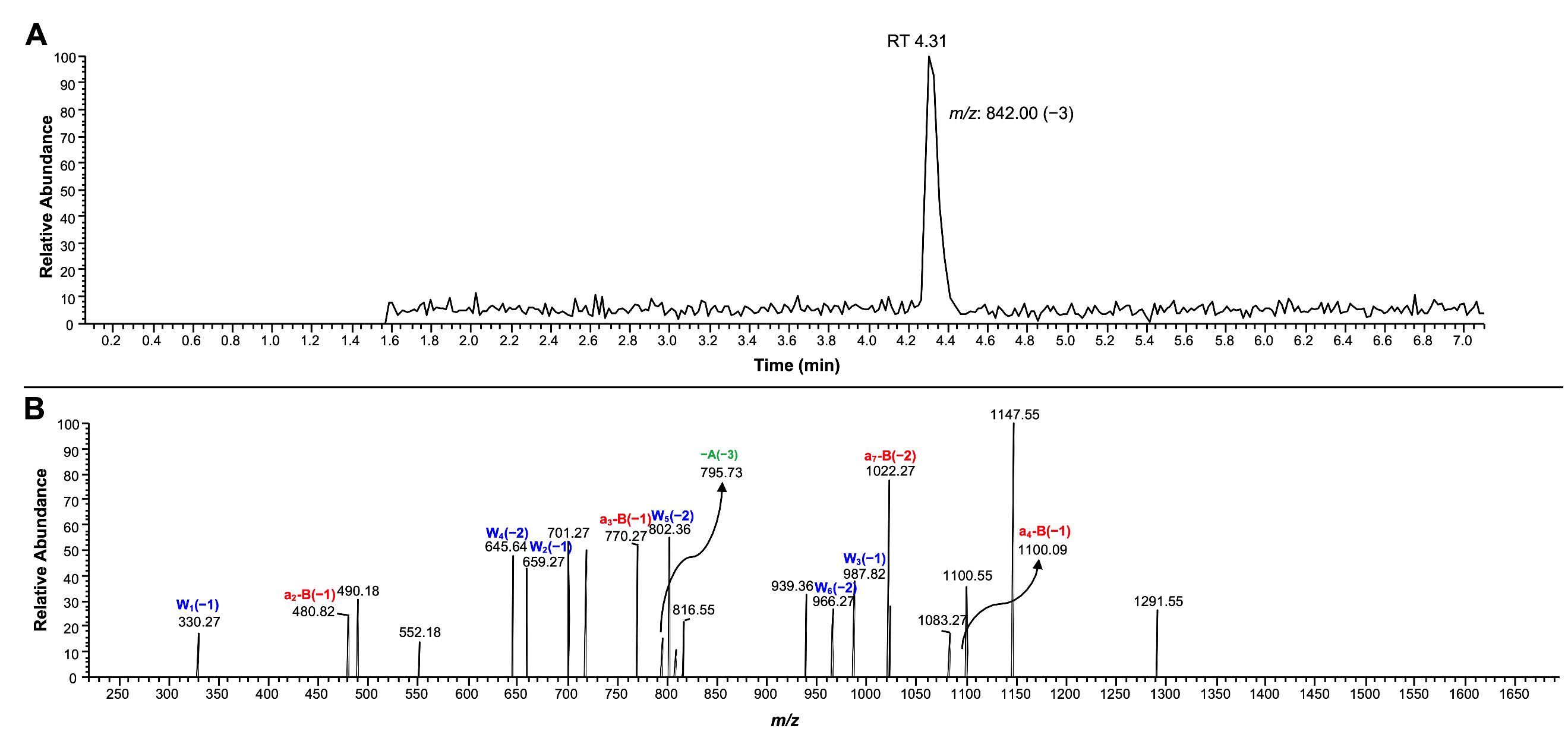


**Fig. S18. LC-MS/MS sequencing analysis of full-length extension reactions for 1,*N*^2^-ε-G modified** **template 2 (5´-T(εG)G-3´) and Primer_8.** *A*, extracted ion chromatogram for *m/z* 842.00 (−3, *t*_R_ 4.31 min) for extended product sequence 5′-pTC**G**ATG**G**A-3′; *B*, CID spectrum of *m/z* 842.00 (−3). See Table S10 for fragment assignment.

**Table S10**

**Observed and theoretical CID fragments of *m/z* 836.64 (−3) from full-length extended products for 1,*N*^2^-ε-G modified** **template 2 (5´-T(εG)G-3´) and Primer_8**

The extended product sequence is 5′-pTC**G**ATG**G**A-3′ (Fig. S18*B*), indicating insertion of G, misinsertion of G at 5´T, plus blunt end addition of A.

| **Fragment assignment** | ***m/z* observed** | ***m/z* theoretical** |
| --- | --- | --- |
| 5′-pTC (a_2_-B, −1) | 480.82 | 481.27 |
| 5′-pTCG (a_3_-B, −1) | 770.27 | 770.45 |
| 5′-pTCGA (a_4_-B, −1) | 1100.09 | 1099.66 |
| 5′-pTCGATGG (a_7_-B, −2) | 1022.27 | 1022.63 |
| pGATGGA-3′ (W_6_, −2) | 966.27 | 967.12 |
| pATGGA-3′ (W_5_, −2) | 802.36 | 802.51 |
| pTGGA-3′ (W_4_, −1) | 645.64 | 645.91 |
| pGGA-3′ (W_3_, −1) | 987.82 | 988.63 |
| pGA-3′ (W_2_, −1) | 659.27 | 659.42 |
| pA-3′ (W_1_, −1) | 330.27 | 330.27 |


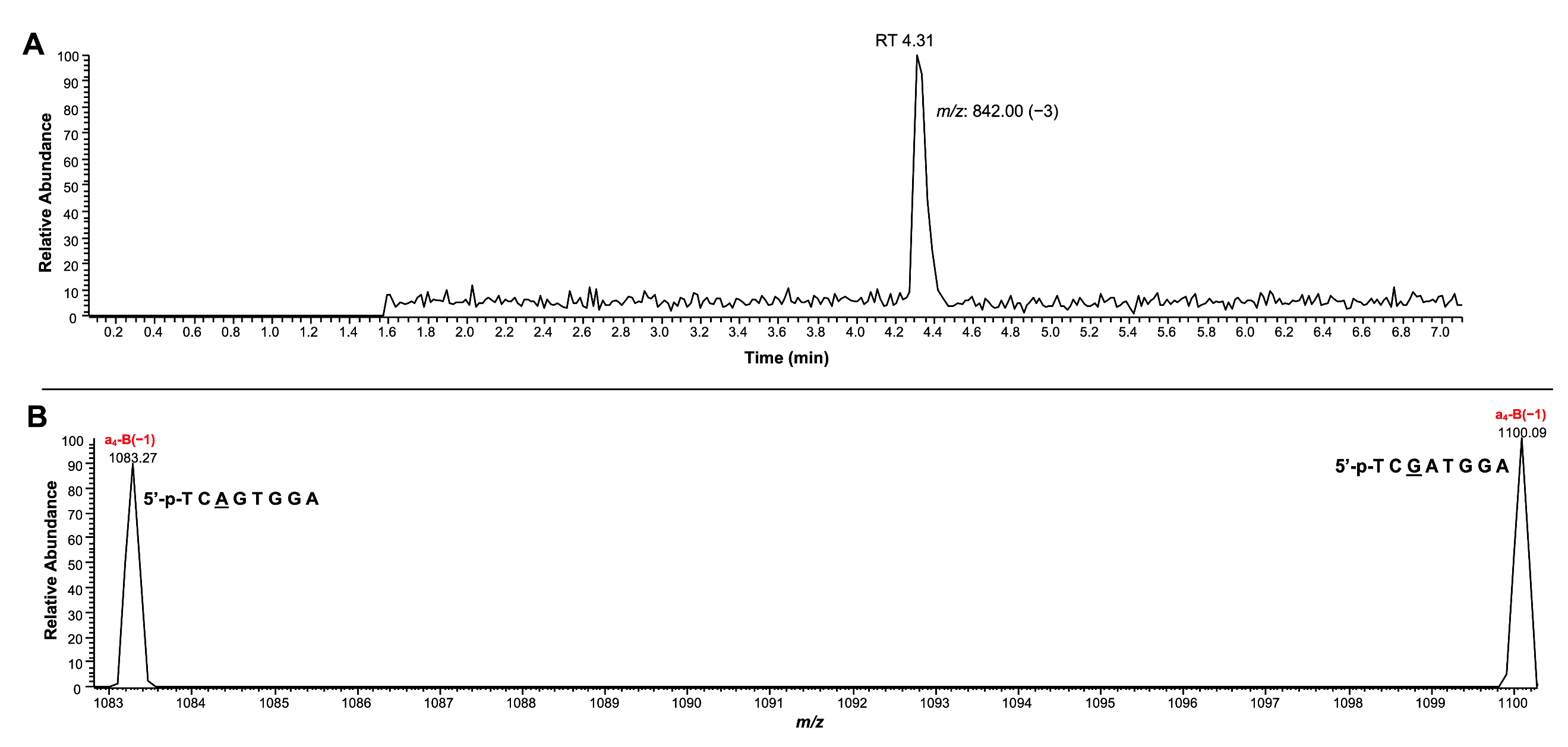


**Fig. S19. LC-MS/MS sequencing analysis of full-length extension reactions for template 2 (5´-T(εG)G-3´) and Primer_8.** *A*, extracted ion chromatogram for *m/z* 842.00 (−3, *t*_R_ 4.31 min); *B*, expanded region of CID spectrum of *m/z* 842.00 (−3), a representative a_4_-B(−1) fragment from each product is shown.


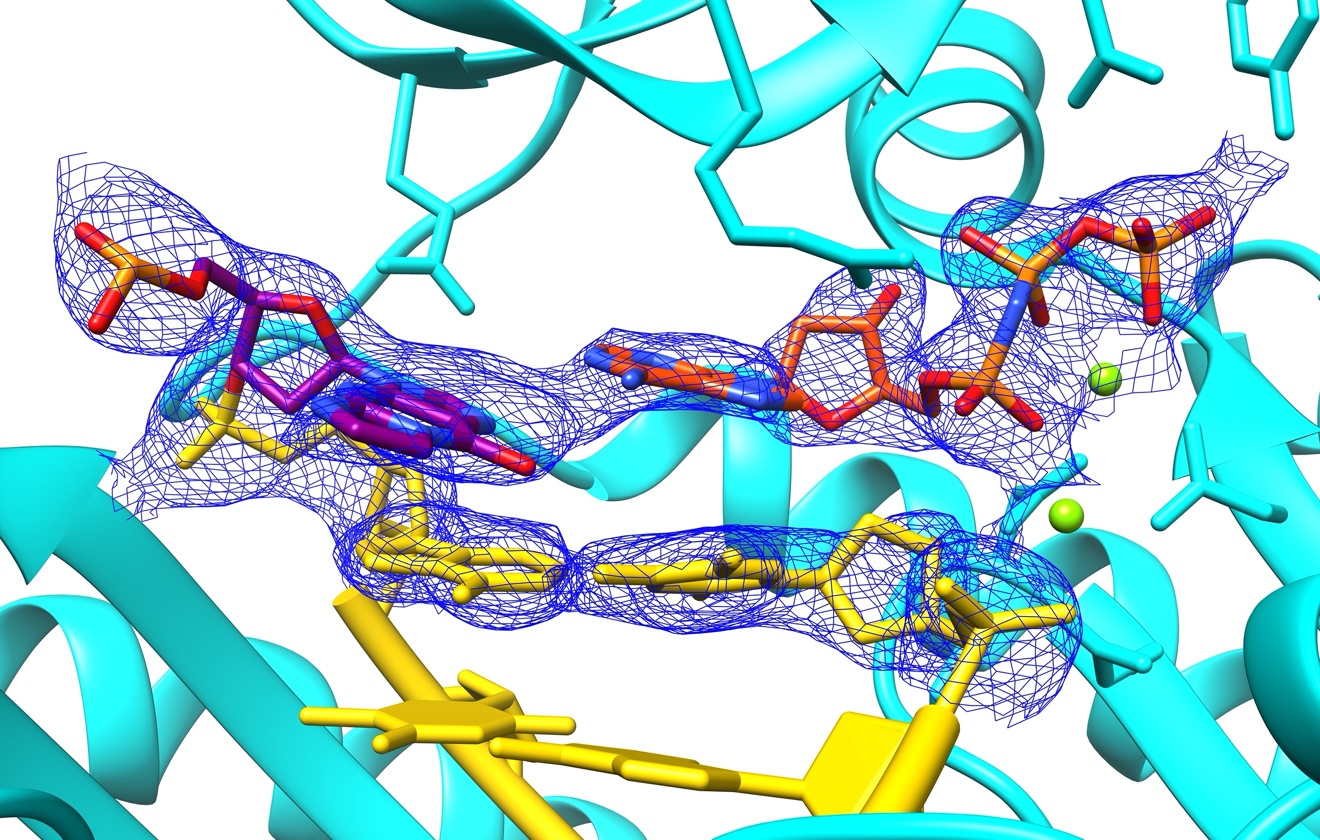


**Fig. S20**. Quality of the final Fourier 2Fo-Fc sum electron density (1σ threshold) in the active site region of the ternary complex between hpol η:1,*N*^2^-ε-G-adducted template-primer duplex and dAMPnPP.


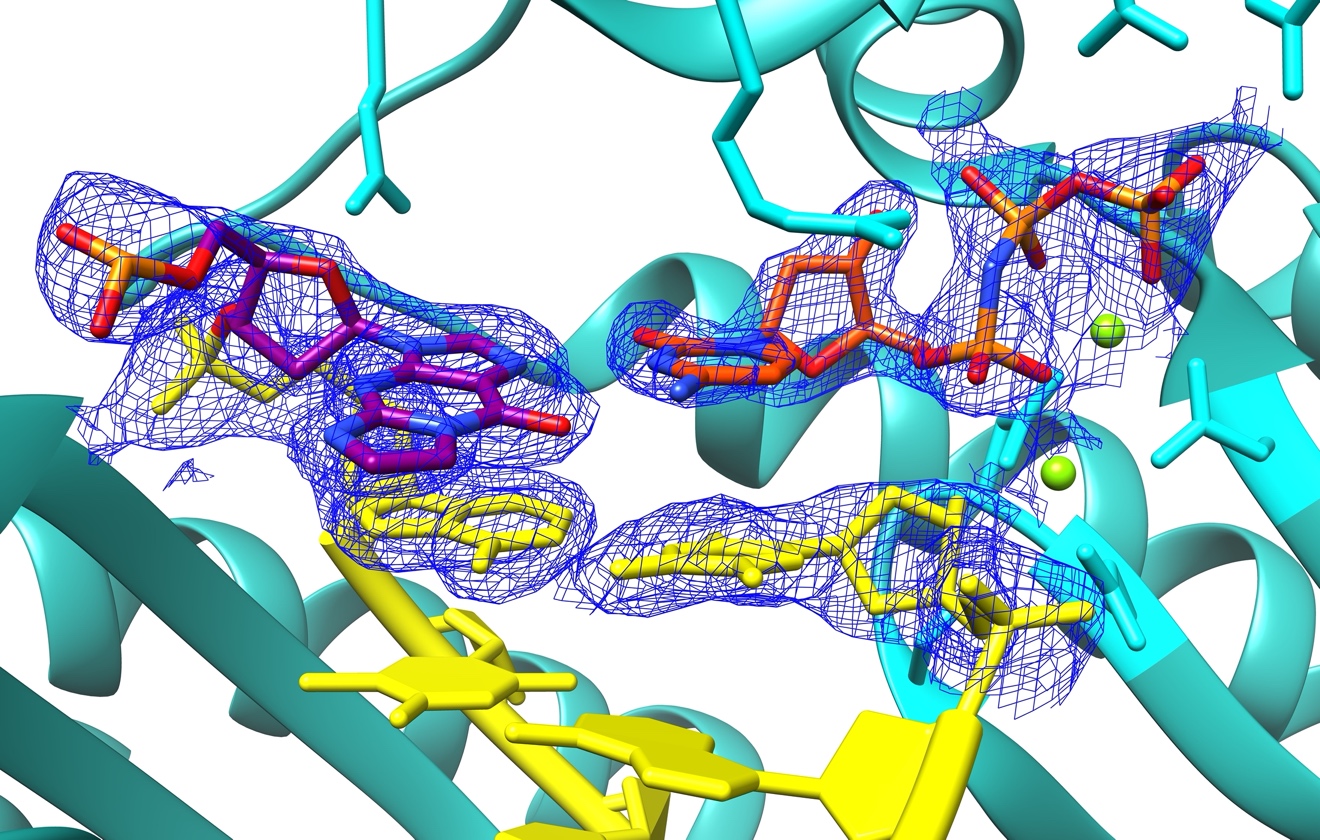


**Fig. S21**. Quality of the final Fourier 2Fo-Fc sum electron density (1σ threshold) in the active site region of the ternary complex between hpol η:1,*N*^2^-ε-G-adducted template-primer duplex and dCMPnPP.
